# Family Planning in the Democratic Republic of the Congo: Encouraging Momentum, Formidable Challenges

**DOI:** 10.9745/GHSP-D-17-00346

**Published:** 2018-03-21

**Authors:** Dieudonné Kwete, Arsene Binanga, Thibaut Mukaba, Théophile Nemuandjare, Muanda Fidele Mbadu, Marie-Thérèse Kyungu, Perri Sutton, Jane T Bertrand

**Affiliations:** a Government of the Democratic Republic of the Congo (DRC), Advisor to the Prime Minister, Kinshasa, DRC.; b Tulane International LLC, Kinshasa, DRC.; c United States Agency for International Development/DRC, Kinshasa, DRC.; d United Nations Population Fund, Kinshasa, DRC.; e Programme National de Santé de l'Adolescent, Ministry of Health, Kinshasa, DRC.; f Programme National la Santé de la Reproduction, Kinshasa, DRC.; g The Bill & Melinda Gates Foundation, Seattle, WA, USA.; h Tulane School of Public Health and Tropical Medicine, New Orleans, LA, USA.

## Abstract

Momentum for family planning in the Democratic Republic of the Congo (DRC) is evident in multiple ways: strong political will, increasing donor support, a growing number of implementing organizations, innovative family planning programming, and a cohesive family planning stakeholder group. Between 2013 and 2017, the modern contraceptive prevalence rate (mCPR) in the capital city of Kinshasa increased from 18.5% to 26.7% among married women, but as of 2013–14, it was only 7.8% at the national level. The *National Multisectoral Strategic Plan for Family Planning: 2014–2020* calls for achieving an mCPR of 19.0% by 2020, an ambitious goal in light of formidable challenges to family planning in the DRC. Of the 16,465 health facilities reporting to the national health information system in 2017, only 40% offer family planning services. Key challenges include uncertainty over the political situation, difficulties of ensuring access to family planning services in a vast country with a weak transportation infrastructure, funding shortfalls for procuring adequate quantities of contraceptives, weak contraceptive logistics and supply chain management, strong cultural norms that favor large families, and low capacity of the population to pay for contraceptive services. This article describes promising initiatives designed to address these barriers, consistent with the World Health Organization's framework for health systems strengthening. For example, the national family planning coordinating mechanism is being replicated at the provincial level to oversee the expansion of family planning service delivery. Promising initiatives are being implemented to improve the supply and quality of services and generate demand for family planning, including social marketing of subsidized contraceptives at both traditional and non-traditional channels and strengthening of services in military health facilities. To expand contraceptive access, family planning is being institutionalized in nursing schools, allowing students to operate as community-based distributors. While major challenges remain, significant progress in family planning has been made in the DRC, which should be judged not in comparison with sub-Saharan African countries with high mCPR and mature programs, but rather with those starting from much further behind.

## Abstract (precis)

**Formidable challenges:** uncertain political situation, cultural norms favoring high fertility, a thin patchwork of service delivery institutions, logistical issues in a vast country with weak infrastructure, and low capacity of the population to pay for contraceptive services. **Encouraging progress:** increasing government and donor support, openness to progressive service delivery policies, innovative programming including robust social marketing and initiatives with nursing schools and the military, strong collaboration among stakeholders, high unmet need suggesting strong latent demand for family planning, and an increasingly balanced method mix including long-acting methods.

See also the French version of this article.

## BACKGROUND

The Democratic Republic of the Congo (DRC), with a population of 79,723,000 in 2016, ^1^ is the third largest country in sub-Saharan Africa and the largest francophone country in the region. The total fertility rate increased slightly between the 2007 and 2013–14 Demographic and Health Surveys (DHS), from 6.3 to 6.6 children per woman.^2^^,^^3^ The increase in the modern contraceptive prevalence rate (mCPR) for married women between these surveys was minimal: 5.8% to 7.8%. Similar to the majority of sub-Saharan African countries, cultural norms favor large families, with fertility rates higher in rural than urban areas.

Although the country had a promising, fledging family planning program in the 1980s (*Projet des Services des Naissances Desirables*) supported by the United States Agency for International Development (USAID), the political turmoil and economic devastation that gripped the country for over a decade starting in 1991 and the subsequent all-African War in 1998 virtually obliterated this earlier progress. The donor community withdrew technical and financial support for family planning, which did not resume until the mid-2000s. Problems related to fiscal mismanagement in governance led to minimal investment of less than 1% of gross domestic product (GDP) in health,^4^ of which little went to family planning. Development programs in general and family planning programs in particular were paralyzed until international donors, including USAID, the United Nations Population Fund (UNFPA), and the Department for International Development (DFID), cautiously returned to the DRC to fund the health sector in the mid-2000s. As the DRC began a slow return to normalcy after the 2006 elections, the donors were reluctant to invest in a country with weak program leadership, poor managerial systems, and little political support for family planning.

A few stakeholders in family planning organizations, such as Population Services International (PSI), Association pour le Bien-Etre Familial–Naissances Désirables (ABEF-ND) (a member association of the International Planned Parenthood Federation), and SANRU (Santé Rurale), were able to maintain a presence in the DRC during the lost decade of the 1990s, despite geographically limited and uncoordinated interventions; however, the current configuration of family planning actors and organizations is relatively new. Two government entities—the National Program for Reproductive Health (Programme National de Santé de la Reproduction, or PNSR) and the National Program for Adolescent Health (Programme National de Santé de l'Adolescent, or PNSA)—have the mandate to guide policy and establish norms for family planning and sexual/reproductive health (SRH), respectively, but they do not have the human or financial resources to deliver family planning services on a national scale. Moreover, no single international donor has the resources or inclination to support the rebuilding of a national family planning program that would extend family planning coverage to all 516 health zones in this vast country. The country's health policy is based on primary health care, of which family planning is one of the components. Donors who work alongside the Government of the DRC support primary health care activities in the health zones.

No single international donor has the resources or inclination to support the rebuilding of a national family planning program in DRC.

As the large international donors returned to the DRC in the mid-2000s, USAID, the World Bank, and DFID opted to support integrated health services delivery in a subset of the country's 516 health zones, largely in rural areas. Currently the USAID-funded Integrated Health Project (Project de Santé Intégré, or PROSANI) operates in 126 health zones; the DFID-funded Access to Primary Health Care Project (Projet d'Accès aux Soins de Santé Primaire, or ASSP) supports service delivery in 52 health zones. UNFPA's main role is to procure contraceptives for government programs and international NGOs; to a lesser degree, it funds specific family planning service delivery interventions in selected locations. Since 2013, UNFPA has directly supplied 63 health zones with family planning commodities and indirectly supported 150 additional health zones through its contraceptive procurement for at least 6 implementing organizations. The World Bank also supports integrated health service delivery through a pay-for-performance mechanism; its current project—which became operational in May 2016—will eventually cover an additional 169 health zones, of which 105 have no other external support for family planning. By contrast, the Bill & Melinda Gates Foundation and the David and Lucile Packard Foundation opted to invest in family planning services in the capital city of Kinshasa, and began support in 2011 and 2013, respectively. The map in the Figure shows health zones that receive assistance for family planning from 1 or more external partners.

**FIGURE:**
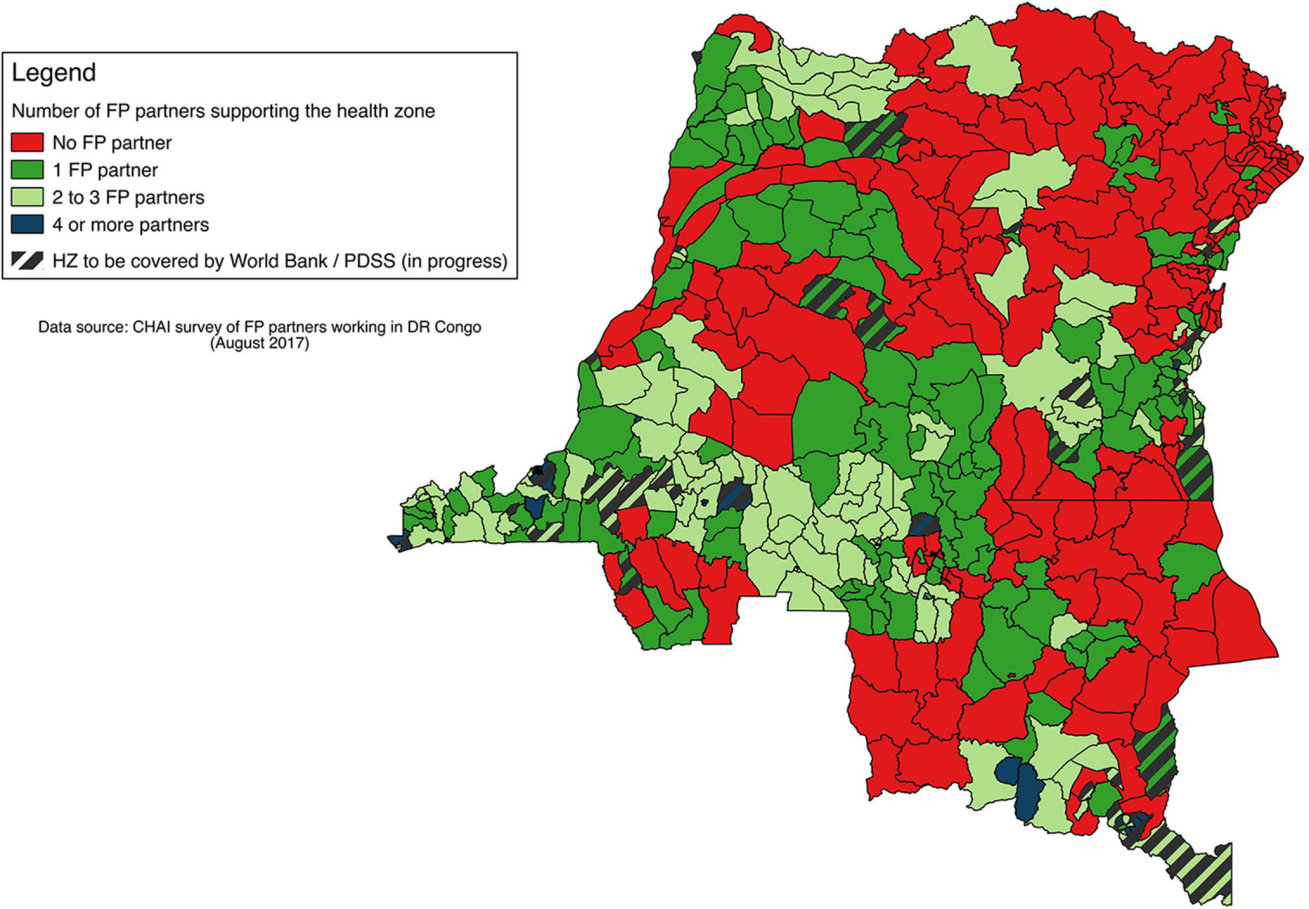
Map of Health Zones in the DRC Receiving Family Planning Assistance Abbreviations: CHAI, Clinton Health Access Initiative; DRC, Democratic Republic of the Congo; FP, family planning; HZ, health zone; PDSS, Health System Strengthening for Better Maternal and Child Health Results Project. Source: Clinton Health Access Initiative.^5^ Map prepared by Dr. Julie Hernandez.

As a result, the approach to family planning service delivery in the DRC is best described as piecemeal. Health zones that benefit from the support of one or more external donors are far more likely to have a range of modern contraception and trained personnel available than others; a study in Kinshasa demonstrated that health facilities receiving external family planning support had higher levels of output than those that did not.^6^ Moreover, each health zone is subdivided into *aires de santé* (health areas). Even the large-scale donor-funded integrated health projects may cover only a portion of the *aires de santé* in a given health zone. As of mid-2017, only two-thirds (66.4%) of the 516 health zones in the DRC received any external assistance for family planning.^5^ (The health zones that the World Bank project is expected to cover are shown separately in the Figure, because as of mid-year 2017 contraceptive commodities and services were not yet available in all World Bank-supported health zones. Once realized, the percentage of health zones receiving external assistance for family planning will increase to 75.9%.)

As of mid-2017, only two-thirds of the 516 health zones in the DRC received any external assistance for family planning.

The mechanisms for family planning service delivery differ by province and by health zone, largely influenced by support from an external donor. The provinces of Kinshasa and Nord Kivu have the greatest concentration of family planning services: the former because it is the capital city that represents 14% of the population of the country; the latter because of intense investment by humanitarian organizations. By contrast, largely rural provinces with little or no external family planning support (shown in red in the Figure) may lack family planning services, even in the hospitals and health centers of the major towns. According to a study conducted in 2014, only 33% of health facilities in the DRC have family planning available, and of these only 20% provide quality services according to criteria specific to the study.^7^

The use of contraception in the DRC is characterized by 2 relatively unusual trends. First, as of the 2013–14 DHS, the use of traditional methods (rhythm, withdrawal, other) was higher (12.6%) than the use of modern methods (7.8%) among married women.^3^ Second, as a percentage share among all married users of modern contraception, the male condom (43.6%) was by far the leading method, followed by injectables (15.4%) and female sterilization, pills, and implants (each at 9.0%).^3^ The only other country in sub-Saharan Africa in which traditional method use is higher than modern method use is Congo-Brazzaville (just across the Congo River from Kinshasa).^8^ The male condom is the leading modern contraceptive method in only 18% of sub-Saharan African countries.^9^ Several factors may explain the high reliance on traditional methods: the years of political turmoil that led to a scarcity of all material goods, the deeply ingrained fear of side effects—particularly sterility—from modern methods, and the promotion of natural methods by faith-based organizations that run more than half of the health facilities in the country. The high use of male condoms may reflect their availability—free or at very low cost—thanks to widespread distribution by HIV prevention programs.

Use of traditional methods (12.6%) is higher than use of modern methods (7.8%) among married women in the DRC.

The commercial private sector (pharmacies and drug shops) represents the leading source of contraception among users in all recent surveys (DHS, Performance Monitoring and Accountability 2020 [PMA2020]), in large part because condoms have been the most widely used modern method. By contrast, users of female sterilization, implants, and injectables look to fixed facilities: government or private hospitals or health centers.^3^ Two large social marketing groups, PSI and DKT International, provide pills, condoms, emergency contraception, and the subcutaneous depot medroxyprogesterone acetate (DMPA-SC) injectable, known locally under the brand name Sayana Press, through commercial outlets at subsidized prices, primarily in urban areas. Contraceptives are available through a very limited number of pharmacies in major urban areas at the full non-subsidized price, but few users can afford to purchase methods through these outlets, and pharmacies make little profit from these sales. Also, no contraceptives are manufactured in the DRC; all are imported, either through international mechanisms (e.g., USAID, UNFPA, and the International Planned Parenthood Federation) or purchased directly from manufacturers (e.g., in Germany, India, Malaysia, and Thailand). “The category of ‘contraception’ is underdeveloped or non-existent in pharmacies in the DRC,” according to one marketing specialist (personal communication, Jacques-Antoine Martin, 2018). “Products are hidden behind counters, except for condoms, and clients ‘whisper’ about contraception if they come to purchase it.”

Community-based distribution takes 2 forms. In the large-scale rural health programs (PROSANI, ASSP), community health workers known as *relais communautaires,* provide pills and condoms at the community level. In Kinshasa and Kongo Central, the AcQual II project has established a network of more than 1,000 community-based distribution workers with a nonmedical profile to provide pills, condoms, and CycleBeads (a tool that helps a woman identify and count her fertile days using the Standard Days Method of family planning).

From 2000 to 2012, the DRC worked without a clear achievable family planning objective. The DRC government began to express interest in family planning around the time of the 2012 London Summit on Family Planning. Members of the government and other family planning stakeholders channeled this interest into the *National Multisectoral Strategic Plan for Family Planning: 2014–2020*, which established the objective of increasing mCPR for all women of reproductive age from 6.5% (in 2013) to 19.0% by 2020.^10^ Can this be done in a vast country that does not have a national program with the necessary financial and human resources to ensure contraceptive access in both urban and rural areas? Several strategies show promise despite the seemingly insurmountable barriers.

## ENCOURAGING DEVELOPMENTS

### Political Will for Family Planning

During the first decade of the 2000s, several organizations offered family planning services, especially in Kinshasa, but they did so in the absence of strong leadership or a clear vision for family planning. The first sign of renewed interest in this topic emerged in 2009 with the Second National Conference on Repositioning Family Planning, which was held under the auspices of the First Lady. An important recommendation of the conference was to establish a key stakeholder group—the Technical Multisectoral Permanent Committee (Comité Technique Multisectoriel Permanent, or CTMP)—to guide future family planning initiatives in the country. Due to a lack of resources and organizational mechanisms, this committee existed but remained relatively inactive for several years.

As previously published in this journal,^11^ the government of the DRC gave little priority to the issue of family planning prior to 2012. However, a series of positive events began in 2012 that have resulted in strong political support for family planning on the part of the government, emanating from the office of the Prime Minister. At a local conference held in June 2012 as a call to action for increased family planning funding, the Ministry of Health pledged to mobilize government funding for family planning. Consistent with growing support for family planning, the Prime Minister signed an official letter to the Minister of Planning in 2013, mandating greater engagement in family planning. The second *Growth and Poverty Reduction Strategy Paper 2011–2015*^12^ highlights development strategies, including the need for access to reproductive health and family planning services.

In 2013, the DRC government publicly pledged commitment to family planning at the Third International Conference on Family Planning in Addis Ababa, Ethiopia.^13^ Shortly thereafter, the government launched the *National Multisectoral Strategic Plan for Family Planning: 2014–2020.*^10^ For the first time ever, in 2014 the government paid $300,000 for contraceptive procurement. In December 2014, it also pledged an additional $2.5 million during the round table at the Third National Conference on Repositioning Family Planning. In December 2015, the National Assembly approved a line item of $3.5 million in the national budget. In December 2016, the DRC government disbursed $1 million for the purchase of contraceptives. The DRC was the only country to send 3 ministers (Health, Education, and Plan) to the Fourth International Conference on Family Planning in Nusa Dua, Indonesia, where they presided over several panels. In addition, the Prime Minister of the DRC delivered a speech via video at the closing plenary session of this conference.

PNSA has also emerged as an important force in the DRC. In 2015, it organized a round table on the sexual and reproductive health of adolescents and youth, which gave more visibility and cohesion to partners working in this field. The following year, PNSA brought together donors and implementing partners to develop and publish the *National Strategic Plan for the Health and Well-Being of Adolescents and Youth: 2016–2020*,^14^ one of the first of its kind in a sub-Saharan African country.

### Increased Donor Investment

Encouraged by these clear signs of political will in the DRC, the number of donors investing in family planning in the DRC has increased markedly over the last 5 years, as has the dollar amount of their investments. Whereas several major donors (e.g., USAID, DFID, the World Bank) continue to focus their support on large-scale integrated health delivery projects in rural areas, more recently donors have tended to support new approaches to service delivery, test innovative strategies, and fund adolescent/youth programs with an emphasis on urban areas.

Encouraged by clear signs of political will in the DRC, the number of donors investing in family planning in the country has increased markedly over the last 5 years.

Examples of major new investments include two 5-year programs that will extend coverage and reduce the financing gap for contraceptive procurement: the Global Financing Facility (GFF) under World Bank leadership and the Central Africa Forest Initiative (CAFI)/Reducing Emissions from Deforestation and Forest Degradation (REDD+) through the Norwegian Agency for Development Cooperation (Norad). In 2015 the DRC was selected among the focus countries for the GFF, a new initiative launched in 2015 to advance reproductive, maternal, newborn, child, and adolescent health.^15^ The country investment case developed under the GFF lists family planning among the priority interventions that will be implemented in 14 selected provinces showing the poorest track records in maternal, newborn, child, and adolescent health. CAFI/REDD+ is an environmental project that includes a family planning component in recognition of the role that population growth plays as one of the drivers of rapid deforestation in the Congo basin. In addition, 2 anonymous donors have begun to invest in both service delivery and demand creation activities in the DRC in recent years. Starting in 2016, the government of the Netherlands joined the list of international family planning donors, with support to youth programming in the 2 Kivu provinces under the title *Jeunes S3 (Sécurité, Santé et Sexualité*) and contraceptive supply chain management (2016–2020).

### Climate of Innovation

The DRC has often lagged behind other countries in the region in terms of innovation and experimentation in other areas of public health, in part for the reasons cited above of limited financial and human resources. Yet in family planning, the DRC has been a hub for innovation. In 2012 the website, www.familyplanning-drc.net (also available in French at www.planificationfamiliale-rdc.net), was the first of its kind to provide information on a large range of family planning topics in the DRC. In 2013, the DRC was the second country selected to participate in the PMA2020 survey program, an innovative mechanism for collecting population and facility-based data: interviewers who reside in the “enumeration area” collect data via smartphones that can be transmitted directly to a server for timely analysis.^16^ In 2015, a pilot study on the introduction of DMPA-SC demonstrated the acceptability and feasibility of having medical/nursing students provide this method (among others) at the community level.^17^ Efforts are underway to expand the use of the DHIS 2 platform as a routine health information system to analyze contraceptive availability throughout this immense country, incorporate geographic information system (GIS) data that will preclude the need for separate mapping exercises, and use the DHIS 2 as a complementary logistics management information system.

As of 2014, little programming focused on the sexual and reproductive health problems of adolescents and young people. Only 20 of the 516 health zones offered SRH services to adolescents and youth of all types. Yet the climate for youth programming changed shortly after the Third National Conference on Repositioning Family Planning in December 2014. PNSA worked with its technical partners to develop a package of SRH services, including both a clinical and outreach component. A round table on sexual and reproductive health among adolescents and young people, held in December 2015, showcased the fledgling but growing project activity in this area. In 2016, PNSA spearheaded the development of a strategic plan for this population.^14^ The Bill & Melinda Gates Foundation, the David and Lucile Packard Foundation, and the governments of Sweden and the Netherlands are investing in programs that focus on adolescents and youth. UNFPA provided contraceptives to these programs. In 2017, PNSA with support from the United Nations Children's Fund (UNICEF) developed a listing of all SRH services for youth and adolescents in the DRC, which indicated 120 health zones receiving support for this type of service. In short, support for SRH programming for adolescents and young people in the DRC—though still in its infancy—has risen steadily in a period of 24 months.

Support for sexual and reproductive health programming for adolescents and young people in the DRC has risen steadily over a period of 24 months.

### Unifying Mechanism for the Family Planning Community: The CTMP

Following the 2009 recommendation resulting from the Second National Conference on Repositioning Family Planning, the CTMP began functioning as a coordinating entity in 2012. Its organizational members included several ministries (Health, Plan, and Gender), donors, and international and local NGOs. Representatives from different donor organizations and international NGOs realized the benefits of this mechanism in planning for the local family planning conference in 2012. As family planning activity accelerated in 2013 with the prospect of the DRC publicly pledging at the Addis Ababa International Conference on Family Planning and the development of a national strategic plan, the CTMP took on new relevance. The monthly meetings focus on an array of issues, often depending on current issues (e.g., the Reproductive Health Law, participation in international conferences). They provide a forum for new implementing partners to quickly integrate into the larger community and for visiting donors to meet with family planning stakeholders in a single location. Although this type of coordination mechanism is by no means unique to the DRC, it has given an identity to members of the family planning community, which is then able to “speak as one” on given issues. The Prime Minister's office recognized the value of this group and issued a decree in March 2015, conferring official status to this entity.

### Evidence of Change in Modern Contraceptive Prevalence in Kinshasa

Two DHS surveys conducted in 2007^2^ and 2013–14^3^ showed very little increase in mCPR (from 5.8% to 7.8% among married women of reproductive age) at the national level. The PMA2020 surveys, limited to the capital city of Kinshasa (with the addition of Kongo Central since July 2016), showed an increase in mCPR from 18.5% in 2013 to 26.7% in mid-2017 among women who were married/in union, and from 31.0% to 39.6% among sexually active unmarried women in Kinshasa.^18^^,^^19^ During this same period, the share of long-acting reversible methods among married women using modern methods increased from 10.8% to 40.0% (due almost entirely to use of implants).^18^^,^^19^ As of 2017, unmet need for family planning ranged from 22% to 25% for these 2 groups of women, suggesting strong latent demand for modern contraception. Compared with mCPR in some other sub-Saharan African countries, mCPR remains low. However, the steady increase in contraceptive use and the shift toward long-acting methods in Kinshasa in recent years is promising.

In the capital city of Kinshasa, mCPR among married women increased from 18.5% in 2013 to 26.7% in 2017.

## FORMIDABLE CHALLENGES

Much has been written about the generally weak public-sector health systems in many sub-Saharan African countries: poor infrastructure, lack of basic equipment and supplies, low wages, insufficient training and supervision of personnel, weak information systems, frequent stock-outs of medications, and rude or culturally insensitive treatment of clients by providers, among others.^20^^,^^21^ To this list, we add specific challenges that relate to the political situation, as well as supply and demand for contraception in the DRC.

### The Issue of Political Stability

The current president Joseph Kabila came into power in 2001 (after the murder of his father, President Laurent-Désiré Kabila). His constitutionally mandated 2 terms of office ended in December 2016. The absence of elections in November 2016 provoked political tensions and intermittent demonstrations that made headlines in the global press.

In February 2018, new elections were announced to take place on December 23, 2018, with the new president to take office in January 2019. However, the opposition continues to make strident demands that President Kabila step down now, rather than wait until the elections. As such, the announcement of elections has yet to bring political stability to the country. Most international observers have taken a wait-and-see attitude.

Current and potential donors are keenly aware of the political situation in the DRC as they consider future investments in family planning and other sectors. The challenge is to maintain the confidence of donors, pending a return to conditions of political stability expected to follow successful general elections. Most current family planning donors remain strongly committed to the work already underway, but they remain vigilant of the political volatility in the country.

### The Logistics and Management of Service Delivery

The DRC is the largest country in sub-Saharan Africa in terms of land mass, yet the transportation infrastructure is extremely weak. No highway or train system connects East to West; the 1,736 miles of paved road for the entire country consist of 2-lane highways. Several national airline carriers service the major cities throughout the country, but their safety record is poor. (International agencies depend heavily on the United Nations Humanitarian Air Service for domestic flights.) Less than 7% of the population has access to the Internet,^22^ although mobile SIM penetration has now reached 39.5%.^23^

In addition to logistical challenges, the general shortcomings of the public health system hinder the delivery of family planning services. Inadequate human and financial resources to properly train and supervise health care providers and lack of equipment and the deterioration of physical infrastructure, some of which dates from the colonial era (before 1960), are all difficulties encountered by those working in family planning. Management systems are also inadequate, leading to a situation where health care providers set the prices of their services as they wish, rather than at established standardized prices. Contraceptives provided free of charge in the public sector are often leaked to the private sector, where they are sold for a profit by people whose knowledge of their proper use may be limited.

And as if this were not enough, starting in late 2016, sporadic social and political unrest in areas with heightened security risks endanger personnel trying to conduct activities and resupply health facilities.

### Contraceptive Security and Supply Chain Management

Contraceptive security (having the right contraceptives in the right place at the right time at the right cost) requires astute management and sufficient financial resources to complete a complex series of tasks: quantifying (forecasting) contraceptive needs of a given population, estimating the costs of purchase and transport of the commodities, identifying procurement mechanisms, placing orders, clearing merchandise through customs, ensuring delivery to a central warehouse or directly to regional warehouses, ensuring transport to the facilities where clients will obtain the contraception, tracking the continuous flow of commodities through the system, accounting for all expenses, and financing this entire process.

Multiple factors contribute to the challenges of ensuring contraceptive security in the DRC. Different donors and family planning implementing organizations manage contraceptive procurement and distribution through parallel channels in the national system. Ideally, the supply chain used for essential drugs in the country could integrate contraceptive procurement to improve efficiency and reduce transaction costs. Although discussions are underway to explore this approach, contraceptive procurement and distribution occur outside the government supply chain for essential medicines.

In terms of purchasing contraceptive supplies, the government has used its own funds but is only able to cover a fraction of the costs for a population the size of that in the DRC. Donors are also unable to fill this gap. The logistics of contraceptive distribution—including purchasing, transportation, distribution, and storage—is a major challenge, especially in terms of delivering it to the “last mile” (the client).

The government has used its own funds to procure contraceptives but is only able to cover a fraction of the costs required to meet the needs of the large population.

### The Influence of Sociocultural Norms That Favor Large Families

Demand for family planning services results from the desire to prevent pregnancy in the short term and to space/limit number of births in the long term. All else being equal, societies that favor large families will have less demand for contraception, although such norms evolve over time and in response to macrolevel economic changes (e.g., improved opportunities for women's education, increased urbanization). In the DRC, the total fertility rate is 6.6 children (5.4 in urban areas and 7.3 in rural areas).^3^ As Romaniuk has noted, couples tend not to be deterred from having a large family by their inability to provide for their children in material terms; nor do women limit fertility to participate in the labor force.^24^

Recent qualitative research highlights many factors that contribute to the persistence of high fertility rates.^25^^,^^26^ Having many children is a sign of social status for both men and women. Moreover, Congolese law makes women subordinate to their husbands from the day of their marriage, which gives them less power in their relationship. The husband's family may believe that his wife owes them many children in exchange for the dowry that the husband's family paid for her. If she does not give birth to these children, her husband's family can encourage him to marry another woman. To the extent that parents depend on their children when they become elderly, having many children is a way to ensure that they are taken care of later in life. Finally, children are an important source of labor for families, particularly in rural areas.

Cultural norms can change in response to the economic pressures experienced by families because of their size, particularly in urban areas. However, even women in the highest quintiles for economic well-being in the urban areas of the DRC wish to have an average of 4.9 children, and a higher level of education in the DRC has a minimal effect on the desired family size.^2^

### Fees for Family Planning Services

Per capita GDP in the DRC is $405.^27^ The country ranks 176 of 188 countries on the Human Development Index.^28^ In terms of the percentage of the population living below $1.90 per day, the DRC at 77.1% is one of the most impoverished nations.^27^ In the DRC, 70% of the public health system is financed by user fees, with only 13% covered by the government and 14% by external donors.^29^ This method of direct payment by households constitutes an important financial obstacle to access to care by the poorest segments of the population.

In this climate of grueling poverty, cost can represent a prohibitive barrier to contraceptive use. Programs serving rural areas (e.g., PROSANI, ASSP) provide contraception free of charge. However, registered pharmacies in urban areas charge either market rates or subsidized rates (if they receive commodities from development programs). Hospitals and health centers in urban areas often provide contraception subsidized by the major donors; yet even if the product is low cost or free of charge, the facility may charge for the consultation and supplies required to administer the contraceptive method (e.g., syringes, cotton and alcohol for injections or implant insertions). Social marketing programs, including PSI and DKT International, sell a range of contraceptives at subsidized prices. In addition, PSI, through the Association de Santé Familiale (ASF), instituted a dual system of *payant et gratuité* (paying and free), whereby once a month they supply contraception free of charge, thus affording access to those otherwise unable to pay for the service.

According to FPwatch, an outlet survey conducted in 2015 in Kinshasa and Katanga provinces, the median price per couple-year of protection (CYP) in Kinshasa was $1.75 (and was slightly higher in Katanga).^30^ The cost per CYP was $4.95 for implants and $0.55 for intrauterine devices. Some health facilities in Kinshasa charge between $10 and $20 for the highly popular implant, and a client must pay the full amount of the method on the day of service, even if the cost per year over the multiple years of protection is far lower.

Compounding client inability to pay, prices for contraception are not standardized. PNSR established a list of prices for all contraceptives sold in the public health system, but there is no enforcement of these prices. Moreover, rarely are the prices posted for the benefit of clients. In 2017, only 23% of service delivery points in Kinshasa that charge fees for family planning posted the prices of contraceptives.^19^

## PROSPECTS FOR IMPROVING NATIONAL FAMILY PLANNING COVERAGE

The *National Multisectoral Strategic Plan for Family Planning: 2014–2020* calls for an increase in modern contraceptive prevalence to 19.0% by 2020.^10^ Although PNSR and PNSA serve a normative role and coordinate specific activities, neither has sufficient financial support from government or donors to ensure the provision of family planning services at the national level. Nor is there any prospect on the horizon that either the government or an international donor will come forward with sufficient resources to support a national program that aspires to ensure contraceptive access across this large nation.

Several key initiatives are underway that, if effective, will contribute significantly to improving access to contraception. They align with the 6 pillars of the World Health Organization (WHO) framework for health systems strengthening: governance and leadership, service delivery, personnel, commodities, information, and financing.^31^ The initiatives outlined below are illustrative (not exhaustive) of ongoing work in support of these 6 pillars.

### Governance and Leadership: Transforming the Provincial CTMP Network Into a National Family Planning Alliance

Given the effectiveness of the national CTMP as a coordinating mechanism that provides structure to the family planning community, efforts began in 2016 to establish CTMPs at the provincial level. Of the 26 provinces in the DRC, 12 currently have a provincial CTMP (Kinshasa, Bas Uele, Kasai Central, Katanga, Kongo Central, Lualaba, Nord Kivu, Sankuru, Sud Kivu, Tshopo, Ituri, and Lomani). The Ouagadougou Partnership—a group of 9 francophone West African countries that work together toward a single family planning objective—serves as a useful model for this partnership in the DRC.^32^

A key criterion for establishing a provincial CTMP is the presence of an international NGO that provides family planning services in the province and is willing to play a coordinating role and provide financial support for the organization of CTMP meetings. PNSR remains highly engaged in this initiative. As currently structured, government representatives on the provincial CTMPs include the provincial representative of PNSR, the Provincial Division of Health (Division Provinciale de Santé, or DPS), representatives of at least 3 ministries (Health, Plan, and Gender), and in some cases the ministers of Environment and of Social Affairs, as well as other international and local NGOs.

In the absence of a national program able to provide financial resources for family planning operations at the provincial level, the CTMP constitutes a voluntary grouping of family planning stakeholders interested in advancing family planning at the provincial level. Provincial CTMPs are not operational units; they do not have a budget for program activities. However, these groups of provincial stakeholders will be able to oversee the evolution of family planning service delivery in multiple ways. They are already involved in quantifying contraceptive needs in their provinces. They will have access to rapidly improving service statistics through the adoption of the DHIS2 system in the DRC. These data will allow provinces to track the percentage of health zones (and within them, health areas) that provide family planning services. This type of evidence-based programming is likely to encourage existing funders to fill some program gaps and to attract new investment. Improving the advocacy skills of provincial staff will help secure these gains.

Three key activities in 2017 gave direction to this new initiative. In March, members of the provincial CTMPs met in Kinshasa for the first time, creating solidarity for this type of coordinated approach to promoting family planning in the DRC. In September, PATH organized a 3-day training, attended by 2 persons per province, to strengthen their skills in facilitation techniques using Advance Family Planning (AFP) SMART advocacy tools.^33^ In October, a series of 3 week-long workshops were held (to cover all 12 provincial CTMPs), in which 4 members per province participated in training on the fundamentals of family planning programming, autonomous functioning of the provincial CTMP, improvement of the quantity and quality of family planning coverage, and monitoring progress using service statistics.

Although the provincial CTMPs operate relatively autonomously, the national CTMP will continue to play an important role as a coordinator and catalyst. It will organize annual meetings in which each province will present its achievements and identify areas for improvement, coordinate skills training/workshops at the provincial level, and create a communication platform via a new website (www.ctmp-pf.org).

### Service Delivery: Initiatives to Improve Supply and Demand

The structure of service delivery in the DRC resembles that of most developing countries. Services are available through fixed health facilities (e.g., hospitals, health centers, health posts), pharmacies, and community-based distribution workers. Services are financed through the public sector, private commercial sector, and private not-for-profit (NGO) sector including faith-based organizations. “Hybrid” facilities also exist whereby the government owns the structure and pays the personnel, but an NGO is responsible for training personnel and providing contraceptive commodities.^6^ In addition to registered pharmacies, many informal pharmacies (drug shops) also provide one or more contraceptive methods, such as condoms.

We cite 3 illustrative initiatives designed to increase supply, improve quality, and generate demand. By no means unique to the DRC or innovative on the world stage, they nonetheless represent promising attempts to improve service delivery in the DRC.

#### Social Marketing Programs

Both PSI and DKT International implement social marketing programs in the DRC, which consist of promoting the sale of subsidized contraceptives through multiple channels, both traditional (pharmacies and drug shops) and non-traditional (bars, hotels, gas stations).

The work of DKT International illustrates some of the innovation occurring in the DRC context. The Protected Pleasure Intervention Brigade (*Brigade Intervention de Plaisir Protégé*) operates on the model of a security squad with its “agents” riding in the back of a marked pick-up truck. As the truck pulls up to a bar, the agents (attractive young women and men) dash in and mingle with the customers, promoting the sale of condoms as their “weapon.” In addition to creating brand awareness and destigmatizing contraception, the strategy has demonstrated a willingness for clients to pay. A second strategy involves a network of “bees”: trained nurses, primarily female, who travel to different communities to provide oral contraceptive pills, condoms, and DMPA-SC, targeting low-income women for whom a clinic visit represents a financial barrier. Clients choosing to use DMPA-SC receive a card with the date of their next injection and can opt to receive SMS reminders via cell phone. If a client wants a clinical method, the nurses provide a referral voucher to a facility known to have trained personnel. The nurses receive 50% of the sale of the product, which amounts to $0.50 per DMPA-SC injection. During a 6-month period in 2017, they sold 90,000 units of DMPA-SC.

DKT International is also known as a youth-friendly organization in DRC in approaching young people aged 15 to 24. The NGO has used a human-centered design approach to create a sexual and reproductive health millennial youth program called *Batela Lobi Na Yo* (“Protect Your Future”) to increase uptake of quality family planning products and services among young women in the DRC.

#### Strengthening Family Planning Services Through the Military

Military officials in Kinshasa approached PNSR and others to obtain support for introducing and strengthening family planning service delivery within health facilities run by the military. They also requested a population-based survey to better understand how contraceptive use within the populations living in military camps in Kinshasa differed from the civilian population. The study showed as of 2016, the mCPR of married women living in military camps was much lower (16.0%) than the total population (23.4%).^34^ Because military camps are often considered a “difficult location” to work, previous programs had avoided them. Instead, they are currently partners in programming that trains personnel in fixed facilities, establishes community-based distribution outlets, and features military personnel in billboards promoting family planning in Kinshasa and the adjacent province of Kongo Central. Given the mobility of military personnel in the DRC, it is likely that behavior change within this population will bring benefits to other parts of the country.

#### Generating Demand for Services

Prior to 2015, demand generation consisted primarily of social marketing in major cities and periodic distribution of limited quantities of print materials. Since 2015, several organizations working in behavior change communication (including PNSR), with support from the Johns Hopkins Center for Communication Programs, formed a task force that produced multiple materials: a single, improved logo for use by all family planning organizations; a booklet for low-literacy audiences on the range of available contraceptive methods; and billboards encouraging spousal communication on family planning. This group worked with local TV stations to make family planning a major focus of a “newlyweds” show, and it inserted family planning messages as spots in a popular radio program on health issues. A telephone hotline provides youth and others with a confidential means of obtaining accurate information about contraception. The group also developed and tested a methodology for community-level activities. Such programming is intended to influence behavior at the individual level as well as social norms at the community level.

### Personnel: Institutionalizing Family Planning Through a National Network of Nursing Schools

In 2015 a research pilot on the acceptability and feasibility of having medical and nursing students deliver contraceptives including DMPA-SC at the community level created new awareness for the potential of this cadre as family planning service providers in the DRC.^32^ While the pilot research had set out to test the acceptability of distributing DMPA-SC at the community level, the greatest “discovery” of the study was the potential of using students to expand access to contraception. They are dynamic, well educated, eager to gain experience with service delivery at the community level, and young (thus approachable to young people seeking contraceptives). In addition, they represent tomorrow's leaders of the DRC health care system.^35^

The 6th Directorate (*6ème Direction,* or D6) of the Ministry of Health oversees the 477 schools of nursing and midwifery in the country. (Medical schools operate more autonomously under a different directorate.) In 2016, efforts began within the D6 to institutionalize family planning within nursing schools; specifically, to establish a family planning curriculum that every nursing student will take as the first module of the third academic year; to train instructors from these schools to teach this subject matter; and to incorporate a community-based practicum into the curriculum in which students would provide 5 contraceptive methods (Implanon NXT, DMPA-SC, pills, condoms, and CycleBeads) at the community level. Because of the growing popularity of implants through the DRC, the use of this cadre of provider to administer Implanon NXT outside a clinical setting further enhances the potential effectiveness of this strategy.

To date, D6, PNSR, and other stakeholders have instituted this program in 11 nursing schools in Kinshasa and 6 nursing schools in the adjacent province of Kongo Central. Students are operating as community-based distributors. The National Health Information System (Système National d'Information Sanitaire, or SNIS) is modifying its family planning data collection forms to reflect the contribution of this new cadre of personnel.

Family planning has been institutionalized in 11 nursing schools in Kinshasa and 6 nursing schools in Kongo Central, with students operating as community-based distributors.

This innovative mechanism represents a first step in expanding and strengthening the delivery of family planning services in the DRC. It is by no means the total solution to this enormous challenge. Yet it has emerged as one of the potentially most effective means of jump-starting service delivery, especially where it can expand to provinces beyond Kinshasa.

### Commodities: Ensuring Contraceptive Security Through Improved Supply Chain Management

It is hard to overstate the enormity of the challenge of ensuring contraceptive security in a country that is physically immense, has weak transportation systems, is extremely underfunded for contraceptive procurement, and lacks a unified procurement/distribution system. The DRC can boast little “innovation” in this area. Rather, it must focus on establishing the basic elements of a functional contraceptive logistics system, which is itself an ambitious objective but fundamental to achieving increased mCPR. The enormity of the logistical challenge has caused some to ask if the DRC should be a testing ground for using drones to deliver contraceptive commodities.

Several positive steps in this domain include the following:

- Establishing a multiagency contraceptive logistics task force, including government, international NGOs, and donors
- Quantifying contraceptive needs for the country, by province, from 2016–2020
- Estimating the cost for contraceptive procurement and family planning service delivery, as well as the financial gap that exists to meet these needs
- Establishing an early-warning system for contraceptive stock-outs at the health zone level, with mechanisms to trigger resupply
- Lobbying the country's health donors to allocate a budget for contraceptives

Key activities are either underway or in the planning stage that will contribute to improving the effectiveness of contraceptive logistics:

- Implementing a national strategic plan for the supply chain to improve the alignment and effectiveness of government, donors, and partners (recently signed by the Minister of Health)
- Developing greater cohesion and coordinated action among members of the multiagency contraceptive logistics task force
- Developing an accurate, comprehensive, and transparent information system on contraceptive procurement and distribution of contraceptives flowing into the country for use in the public and private, nonprofit sector
- Maintaining a strong advocacy initiative to mobilize funding among international donors to cover the contraceptive financing gap

In 2016, an initiative began to review the possibility of incorporating contraceptives into a larger government-supported system for procuring and distributing essential commodities. At the heart of the system is a quasigovernmental organization responsible for the procurement of health commodities in the DRC called FEDECAME (Fédération des Centrales d'Approvisionnement en Médicaments Essentiels). FEDECAME is a network composed of a Kinshasa-based procurement and supply chain coordination unit linked to 19 regional distribution centers or *centrales de distribution régionale* (CDR) for medication throughout the country, whose mandate is to ensure the supply of health commodities through the health system down to the community level. FEDECAME has successfully procured many essential medicines but lacks experience in procuring commodities for family planning, HIV/AIDS, and tuberculosis among other programs, primarily due to historical arrangements whereby vertical donor such as the U.S. President's Emergency Plan for AIDS Relief (PEPFAR), The Global Fund to Fight AIDS, Tuberculosis and Malaria, and the President's Malaria Initiative (PMI) ensured procurement of specific commodities.

Yet should the latter continue to be the long-term objective? A reasonable long-term strategy (for example, 10 years) could perhaps rely on a state-owned and publicly owned supply chain, with major intermediate steps to be accomplished, while continuing to receive meaningful help from donors.

### Information: Strengthening Information Systems to Monitor Progress

The significant progress in this area should be judged not in comparison with sub-Saharan African countries with high mCPR and mature programs, but rather with those starting from much further behind. Three types of information useful to monitoring ongoing programs and advancing family planning programing include the routine health information system, population- and facility-based surveys, and special studies.

#### Routine Health Information Systems

The national health information system (SNIS) has made remarkable progress in the collection of service statistics, including for family planning. Starting in 2013, with strong support from the DFID-funded ASSP project, the 5th Directorate (*5ème Direction,* or D5) of the Ministry of Health began to install the DHIS 2 platform in all (then) 11 provinces of the country. As of late 2017, the system reported data from all (now) 26 provinces of the country, with 95% of health zones reporting on family planning activity. (Each health zone is subdivided into health areas, which include one or more health facilities that report to the system. Whereas 95% of health zones report on family planning services, only 71% of health areas and only 40% of the 16,465 health facilities report some level of family planning activity.) The D5 has worked with the national CTMP in revising the family planning data collected by the SNIS. The system yields the data needed to calculate CYP (a widely used indicator of family planning output). Members of the provincial CTMPs are now trained to access these data from the system and interpret them as a means of monitoring progress at the provincial level. Also, the system can be used to identify health zones (and within them, health areas) that do not have functioning family planning services. Work is underway to obtain GPS coordinates for all health facilities in the system, which will allow for additional analysis of access to contraception.

#### Population- and Facility-Based Surveys

Population-based representative surveys provide a snapshot in time of contraceptive use dynamics, including modern contraceptive prevalence, unmet need, method mix, determinants of use, and other factors. Facility-based surveys describe the family planning supply environment available to clients in a given geography. The DRC was the second (of 11) countries to conduct the PMA2020 survey,^16^ which has yielded 6 rounds of data for Kinshasa and 3 for Kongo Central. The results track the 8 percentage-point increase in mCPR and the steady increase in implant use in Kinshasa over a 5-year period. A second facility-based study, FPwatch, conducted in Kinshasa and Katanga in 2015, provided further in-depth information on the supply environment in these 2 provinces. The next DHS, anticipated in 2019 or 2020, will indicate progress in other provinces and nationally.

#### Special Studies

A series of research pilots conducted by Tulane University in collaboration with local partners have tested the acceptability and feasibility of new strategies for increasing access to contraception at the community level. The first pilot in 2015 tested the use of medical/nursing students to administer DMPA-SC (along with pills, condoms, and CycleBeads).^35^^,^^36^ The positive results led to 3 additional pilots in Kinshasa. The first two, conducted in Kinshasa, tested the acceptability and feasibility of using this same cadre of student to provide Implanon NXT and to teach women in the community to self-inject DMPA-SC. The third, in the rural province of Lualaba, trained *relais communautaires* (non-medical community health workers) to inject DMPA-SC at the community level. In addition, focus group and mystery client studies have indicated the generally favorable attitudes toward emergency contraception and the willingness of pharmacists in official pharmacies to provide emergency contraception to young women.^37^^,^^38^ The use of special studies to continually test new approaches and ideas further contributes to a dynamic family planning environment.

### Financing: Developing the Funding Streams to Cover a Highly Under-Resourced Program

Financing remains a major challenge in a country with few resources and a very small percentage of the national budget allocated to health. Family planning programming in the DRC remains highly donor-dependent, despite the politically important but relatively small contribution of the DRC government to contraceptive procurement. The increased donor investment over the past 5 years shows no signs of slowdown.

At the international level, the GFF is poised to leverage funds from the International Development Association in support of family planning as a priority within the reproductive, maternal, newborn, child, and adolescent health and nutrition continuum. At the national level, several provincial CTMPs—Lualaba, Nord Kivu, and Sud Kivu—have successfully created a line item for contraceptive procurement in the provincial budget.

Efforts to involve the private sector may further contribute to financing family planning service delivery, for example, in the mining sector. Yet if the DRC aspires to universal coverage in family planning by 2030, much work remains to be done to achieve this ambitious goal.

## CONCLUSION

Some have questioned if the norms that favor high fertility in the DRC are likely to change.^26^ Prior to 2012, the government attached little importance to family planning and donors were reticent to invest in family planning in the DRC, given the formidable challenges facing family planning programming. This article provides in-depth analysis of these challenges while highlighting the significant progress made since 2012 in government support and programming of family planning activities in the DRC. It presents promising initiatives expected to contribute to the objective of 19% mCPR by 2020, consistent with the pillars of health systems strengthening. The challenge of procuring sufficient contraceptive supplies to meet the growing demand for contraception is significant, as is the need for improvement in supply chain management to deliver services to the “last mile.” While major challenges remain, this article reflects growing confidence in the ability of government, donors, and implementing partners to influence mCPR, and with it the future of this country.

## Supplementary Material

17-00346-Bertrand-FrenchSupplement.pdf

## Notes

**Peer Reviewed**

**Funding:** The authors are financed by their individual organizations. This article was not commissioned with funding from a single donor.

## References

[1] Country profile: Democratic Republic of Congo. UNdata website. http://data.un.org/CountryProfile.aspx?crName=democratic%20republic%20of%20the%20congo. Accessed May 18, 2017.

[2] Ministère du Plan; Macro International. Enquête Démographique et de Santé, République Démocratique du Congo 2007. Calverton, MD: Ministère du Plan and Macro International; 2008. http://dhsprogram.com/pubs/pdf/FR208/FR208.pdf. Accessed February 1, 2018.

[3] Ministère du Plan et Suivi de la Mise en œuvre de la Révolution de la Modernité (MPSMRM); Ministère de la Santé Publique (MSP); ICF International. Enquête Démographique et de Santé en République Démocratique du Congo 2013–2014. Rockville, MD: MPSMRM, MSP, and ICF International; 2014. http://dhsprogram.com/pubs/pdf/FR300/FR300.pdf. Accessed February 1, 2018.

[4] Barroy H Andre F Mayaka S Samaha H. Investing in Universal Health Coverage: Opportunities and Challenges for Health Finbancing in the Democratic Republic of Congo. Washington, DC: World Bank; 2014. https://openknowledge.worldbank.org/handle/10986/23880. Accessed February 1, 2018.

[5] Clinton Health Access Initiative (CHAI). Outil de cartographie de suivi de distribution des produits PF en RDC. August 21, 2017.

[6] Kayembe P Babazadeh S Dikamba N. Family planning supply environment in Kinshasa, DRC: survey findings and their value in advancing family planning programming. Glob Health Sci Pract. 2015;3(4):630–645. 10.9745/GHSP-D-15-00298. 26681709 PMC4682587

[7] Mpunga D Lumbayi JP Dikamba N Mwembo A Mapatano MA Wembodinga G. Availability and quality of family planning services in the Democratic Republic of Congo: high potential for improvement. Glob Health Sci Pract. 2017;5(2):274–285. 10.9745/GHSP-D-16-00205. 28588047 PMC5487089

[8] Bertrand JT Sullivan TM Knowles EA Zeeshan MF Shelton JD. Contraceptive method skew and shifts in method mix in low- and middle-income countries. Int Perspect Sex Reprod Health. 2014;40(3):144–153. 10.1363/4014414. 25271650

[9] Family Planning 2020 (FP2020). FP2020: The Way Ahead:2016–2017. Washington, DC: FP2020; 2017. http://progress.familyplanning2020.org/en. Accessed February 28, 2018.

[10] Ministère de la Santé Publique (MSP). Planification Familiale: Plan Stratégique National à Vision Multisectorielle (2014–2020). Kinshasa: MSP; 2014. https://advancefamilyplanning.org/sites/default/files/resources/Final%20Plan%20Strategique%20de%20PF%2020%20Fev%20Ok.pdf. Accessed February 1, 2018.

[11] Mukaba T Binanga A Fohl S Bertrand JT. Family planning policy environment in the Democratic Republic of the Congo: levers of positive change and prospects for sustainability. Glob Health Sci Pract. 2015;3(2):163–173. 10.9745/GHSP-D-14-00244. 26085015 PMC4476856

[12] Ministère du Plan. Document de la Stratégie de Croissance et de Réduction de la Pauvreté: Deuxième Génération (2011–2015). Kinshasa: Ministère du Plan; 2011. https://www.afdb.org/fileadmin/uploads/afdb/Documents/Project-and-Operations/RDC_-_2011-2015_-_Document_de_stratégie_de_réduction_de_la_pauvreté.pdf. 2011. Accessed March 23, 2017.

[13] Advance Family Planning (AFP). Democratic Republic of the Congo commits to family planning through the Global FP2020 Partnership. Baltimore, MD: AFP; 2015. http://advancefamilyplanning.org/sites/default/files/resources/drc_EN.pdf. Accessed February 1, 2018.

[14] Ministère de la Santé et de l'Hygiène Publique (MSP); Programme National de Santé de l'Adolescent (PNSA). Plan Stratégique National de la Santé et du Bien-être des Adolescents et des Jeunes 2016–2020. Kinshasa: MSP and PNSA; 2016.

[15] Global financing facility launched with billions already mobilized to end maternal and child mortality by 2030 [news release]. World Bank; July 13, 2015. http://www.worldbank.org/en/news/press-release/2015/07/13/global-financing-facility-launched-with-billions-already-mobilized-to-end-maternal-and-child-mortality-by-2030. Accessed June 14, 2017.

[16] About PMA2020. Performance Monitoring and Accountability 2020 (PMA2020) website. http://pma2020.org/about-pma2020. Accessed Oct 24, 2016.

[17] Hernandez JH Akilimali P Kayembe P Dikamba N Bertrand J. The value of spatial analysis for tracking supply for family planning: the case of Kinshasa, DRC. Health Policy Plann. 2016;31(8):1058–1068. 10.1093/heapol/czw036. 27084735

[18] Performance Monitoring and Accountability 2020 (PMA2020). PMA2013/Kinshasa-R1. Baltimore, MD: PMA2020. https://pma2020.org/sites/default/files/DRC-Kinshasa-R1-EN-FP-Brief-v8-2017-07-13_0.pdf. Accessed February 1, 2018.

[19] Performance Monitoring and Accountability 2020 (PMA2020). PMA2020/Kinshasa, DRC: September-November 2017 (Round 6). Baltimore, MD: PMA2020. https://pma2020.org/sites/default/files/PMA2020-Kinshasa-DRC-R6-FP-Brief-En.pdf. Accessed February 1, 2018.

[20] Patrick Gold O Ufuoma John E. Accelerating empowerment for sustainable development: the need for health systems strengthening in Sub-Saharan Africa. Am J Public Health Res. 2013;1(7):152–158. 10.12691/ajphr-1-7-2

[21] Tumusiime P Gonani A Walker O Asbu EZ Awases M Kariyo PC. Health systems in sub-Saharan Africa: what is their status and role in meeting the health Millennium Development Goals? Africa Health Monitor. 2011;(4). http://www.aho.afro.who.int/en/ahm/issue/14/reports/health-systems-sub-saharan-africa-what-their-status-and-role-meeting-health?width=150&height=150. Accessed February 1, 2018.

[22] Percentage of individuals using the Internet 2000–2016. International Telecommunications Union (Geneva) website. Accessed December 19, 2017.

[23] Democratic Republic of Congo-Telecoms, Mobile and Broadband-Statistics and Analyses. Budde.com website. https://www.budde.com.au/Research/Democratic-Republic-of-Congo-Telecoms-Mobile-and-Broadband-Statistics-and-Analyses. Accessed December 19, 2017.

[24] Romaniuk A. Persistence of high fertility in tropical Africa: the case of the Democratic Republic of the Congo. Popul Dev Rev. 2011;37(1):1–28. 10.1111/j.1728-4457.2011.00388.x. 21714197

[25] Muanda M Gahungu Ndongo P Taub LD Bertrand JT. Barriers to modern contraceptive use in Kinshasa, DRC. PLoS One. 2016;11(12):e0167560. 10.1371/journal.pone.0167560. 27907138 PMC5132197

[26] Mbadu MM Ndongo PG Messina LJ Bertrand JT. Barriers to modern contraceptive use in rural areas in DRC. Cult Health Sex. 2017;19(9):1011–1023. 10.1080/13691058.2017.1286690. 28276915

[27] World Bank. DataBank [database online]. Washington, DC: World Bank; 2017. https://data.worldbank.org/indicator/NY.GDP.PCAP.CD?locations=CD. Accessed February 1, 2018.

[28] United Nations Development Programme (UNDP). Human Development Report 2016: Human Development for Everyone. New York: UNDP; 2016. http://hdr.undp.org/en/2016-report. Accessed February 1, 2018.

[29] World Bank. Congo, Democratic Republic of-health, nutrition and population: country status report. Washington, DC: World Bank; 2005. http://documents.worldbank.org/curated/en/312311468245430956/Congo-Democratic-Republic-of-Health-nutrition-and-population-country-status-report. Accessed May 31, 2017.

[30] Population Services International (PSI); FPwatch. DRC 2015 outlet survey findings. FPwatch Research Brief. Washington, DC: PSI; 2016.

[31] World Health Organization (WHO). Everybody's Business: Strengthening Health Systems to Improve Health Outcomes: WHO's Framework for Action. Geneva: WHO; 2007. http://www.who.int/healthsystems/strategy/everybodys_business.pdf?ua=1. Accessed February 1, 2018.

[32] The Partnership: What's the Ouagadougou Partnership? Planification Familiale: Le Partenariat de Ouagadougou website. https://partenariatouaga.org/en/about-us/the-partnership/. Accessed December 19, 2017.

[33] Advance Family Planning (AFP), Bill & Melinda Gates Institute for Population and Reproductive Health, Johns Hopkins Bloomberg School of Public Health. Develop a Strategy. AFP SMART: A Guide to Quick Wins–Build Consensus, Focus Efforts, Achieve Change. Baltimore, MD: AFP; 2013. https://advancefamilyplanning.org/sites/default/files/2017-07/AFP%20Portfolio%20Component%20Part%202%20EN.pdf. Accessed December 19, 2017.

[34] Akilimali P Anglewicz P Nzuka Engale H. Differences in family planning outcomes between military and general populations in Kinhsasa, Democratic Republic of Congo: a cross-sectional analysis. 2018. Unpublished manuscript. 10.1136/bmjopen-2018-022295 PMC6318504 30580261

[35] Binanga A Bertrand JT. Pilot research as advocacy: the case of Sayana Press in Kinshasa, Democratic Republic of the Congo. Glob Health Sci Pract. 2016;4(4):542–551. 10.9745/GHSP-D-16-00236. 27979874 PMC5199173

[36] Bertrand JT Makani PB Hernandez J. Acceptability of the community-level provision of Sayana Press by medical and nursing students in Kinshasa, Democratic Republic of the Congo. Contraception. 2017;96(3):211–215. 10.1016/j.contraception.2017.05.014. 28647500 PMC5570913

[37] Hernandez JH Mbadu MF Garcia M Glover A. The provision of emergency contraception in Kinshasa's private sector pharmacies: experiences of mystery clients. Contraception. 2018;97(1):57–61. 10.1016/j.contraception.2017.08.001. 28803883 PMC5745145

[38] Hernandez J Mbadu M Garcia M. Knowledge, perceptions and attitudes towards emergency contraception among women of reproductive age women in Kinshasa, DRC. Int Perspect Sex Reprod Health. In press.

