# Supplementary material for: Family Planning in the Democratic Republic of the Congo: Encouraging Momentum, Formidable Challenges
Source: Glob Health Sci Pract. 2018 Mar 21;6(1):40–54. doi: 10.9745/GHSP-D-17-00346 (PMC5878077; doi:10.9745/GHSP-D-17-00346)
Supplement: 17-00346-Bertrand-FrenchSupplement.pdf [file 17-00346-Bertrand-FrenchSupplement.pdf]

GLOBAL HEALTH: SCIENCE AND PRACTICE
Dedicated to what works in global health programs
OPEN ACCESS

REVUE & ANALYSE DE PROGRAMME

La planification familiale en République Démocratique du Congo : Un dynamisme encourageant, des défis impressionnants

Dieudonné Kwete,[a] Arsene Binanga,[b] Thibaut Mukaba,[c] Théophile Nemuandjare,[d] Muanda Fidele Mbadu,[e] Marie-Thérèse Kyungu,[f] Perri Sutton,[g] Jane T Bertrand[h]

Des défis impressionnants : une situation politique instable, des normes culturelles favorisant les familles nombreuses, des institutions de prestation de services fragiles et fragmentaires, des défis logistiques de taille dans un vaste pays aux infrastructures vétustes, et le faible pouvoir d'achat de la population limitant l'acquisition des méthodes de contraception. **Un dynamisme encourageant :** un soutien grandissant du gouvernement et des bailleurs de fonds, une ouverture d'esprit aux politiques de prestation de services progressives, des programmes innovants incluant le marketing social et des initiatives impliquant les étudiants en sciences infirmières et l'armée, une forte collaboration entre les parties prenantes, des besoins non satisfaits élevés suggérant une forte demande latente de planification familiale, et une distribution des méthodes utilisées de plus en plus équilibrée incluant les méthodes à longue durée d'action.

RÉSUMÉ

Le dynamisme de la planification familiale en République Démocratique du Congo (RDC) se reflète par plusieurs aspects, dont : une forte volonté politique, un grand soutien des bailleurs de fonds, un nombre croissant d'Organisations non gouvernementales (ONG) mettant en oeuvre la planification familiale, des programmes innovants et un groupe de parties prenantes clés (le Comité Technique Multisectoriel Permanent, ou CTMP) hautement fonctionnel. Entre 2013 et 2017, le taux de prévalence contraceptive moderne (TPCm) de la capitale du pays, Kinshasa, a augmenté de 18,5% à 26,7% chez les femmes en union, mais en 2013–2014, il n'était que de 7,8% au niveau national. Le *Plan Stratégique National à Vision Multisectorielle de la Planification Familiale : 2014–2020* s'est fixé pour objectif d'atteindre un TPCm national de 19% et de recruter 2,1 millions d'utilisatrices additionnelles à l'horizon 2020, un objectif ambitieux au vu des défis impressionnants qui caractérisent la planification familiale en RDC. Sur les 16 465 formations sanitaires ayant rapporté leurs statistiques de services au Système National d'Information Sanitaire (SNIS) en 2017, seules 40% offraient des services de planification familiale. Nombreuses sont les raisons qui limitent les progrès de la planification familiale en RDC : l'incertitude politique, les difficultés à assurer l'accès aux services dans un pays d'une superficie aussi vaste que l'Europe Occidentale et compte tenu de la vétusté des infrastructures de transport, l'insuffisance des financements pour l'achat des contraceptifs en quantité adéquate, les problèmes liés à la logistique contraceptive et la gestion de la chaîne d'approvisionnement, le poids des normes culturelles qui favorisent les familles nombreuses, ainsi que le faible pouvoir d'achat de la population qui limite l'acquisition des méthodes de planification familiale, sont autant de difficultés qu'il faudra surmonter. Cet article présente des initiatives prometteuses pour surmonter ces obstacles, en accord avec le cadre de travail pour le renforcement des systèmes de santé de l'Organisation mondiale de la santé (OMS). Par exemple, le mécanisme de coordination national pour la planification familiale est en train d'être répliqué au niveau des provinces afin de superviser l'expansion des services de planification familiale. Des initiatives prometteuses sont actuellement à l'oeuvre dans le pays pour améliorer l'offre et la qualité des services, tout en stimulant la demande de planification familiale, dont le marketing social de contraceptifs subventionnés, distribués via des circuits à la fois traditionnels et non traditionnels, et le renforcement des services offerts dans les formations sanitaires de l'armée. Pour étendre l'accès à la contraception, la planification familiale s'institutionnalise dans les écoles de sciences infirmières, permettant aux étudiants d'opérer en tant que distributeurs à base communautaire. Bien que des défis majeurs demeurent, la RDC enregistre des progrès considérables en matière de planification familiale, lesquels devraient être mesurés non pas à l'aune des pays d'Afrique subsaharienne affichant une prévalence contraceptive élevée et dont les programmes sont arrivés à maturité, mais plutôt de ceux qui ont encore beaucoup de chemin à parcourir.

[a] Gouvernement de la République Démocratique du Congo (RDC), Conseiller du Premier Ministre, Kinshasa, RDC.
[b] Tulane International LLC, Kinshasa, RDC.
[c] Agence des Etats-Unis pour le Développement International, Kinshasa, RDC.
[d] Fonds des Nations Unies pour la Population, Kinshasa, RDC.
[e] Programme National de Santé de l'Adolescent, Ministère de la Santé, Kinshasa, RDC.
[f] Programme National de Santé de la Reproduction, Kinshasa, RDC.
[g] Fondation Bill & Melinda Gates, Seattle, Etat de Washington, Etats-Unis d'Amérique.
[h] École de Santé Publique et de Médecine Tropicale de l'Université de Tulane, Nouvelle-Orléans, Etat de Louisiane, Etats-Unis d'Amérique.
Correspondances à Jane T Bertrand (bertrand@tulane.edu).

CONTEXTE

La République Démocratique du Congo (RDC) est le troisième pays le plus peuplé (79 723 000 habitants en 2016[1]) et le plus vaste pays francophone de l'Afrique subsaharienne. Son indice synthétique de fécondité (ISF) a légèrement augmenté entre les Enquêtes Démographiques et de Santé (EDS) de 2007 et 2013-14, passant de 6,3 à 6,6 enfants par femme.[2,3] L'augmentation du taux de prévalence contraceptive moderne entre ces deux enquêtes pour les femmes mariées a été faible, passant de 5,8% (EDS 2007) à 7,8% (EDS 2013–2014). Comme dans la majorité des pays au sud du Sahara, les normes socioculturelles favorisent les familles nombreuses, et l'indice de fécondité en milieu rural est plus élevé qu'en milieu urbain.

Bien que le pays disposait d'un programme de planification familiale prometteur dans les années 1980 (le Projet des Services des Naissances Désirables), soutenu par l'Agence des Etats-Unis pour le Développement International (USAID), les troubles politiques et la crise économique qui s'en est suivie à partir de 1991 et pendant plus d'une décennie, ainsi que la Deuxième Guerre du Congo de 1998, ont anéanti les progrès. La communauté des bailleurs de fonds a alors retiré son appui technique et financier à la planification familiale, lequel n'a repris qu'à la moitié des années 2000. Une gouvernance fiscale inadéquate a conduit à un faible investissement dans la santé : moins de 1% du Produit intérieur brut (PIB),[4] dont très peu de fonds étaient alloués à la planification familiale. Les programmes de développement en général, et de planification familiale en particulier, ont été paralysés jusqu'à ce que les bailleurs de fonds internationaux, dont l'USAID, le Fonds des Nations Unies pour la Population (FUNAP) et le Département du Royaume-Uni pour le Développement International (DFID) reviennent en RDC, non sans précaution, pour financer le secteur de la santé à la moitié des années 2000. Alors que le pays commençait son lent retour à la normalité après les élections de 2006, les bailleurs de fonds étaient réticents à l'idée d'investir dans un pays où le leadership des programmes était faible, les systèmes de gestion précaires, et le soutien politique à la planification familiale quasi nul.

Néanmoins, malgré les périodes troubles des années 1990, quelques intervenants ont continué d'offrir la planification familiale en RDC, dont Population Services International (PSI), l'Association pour le Bien-être Familial-Naissances Désirables (ABEF-ND) (membre de la Fédération Internationale pour la Planification Familiale [IPPF]), et Santé Rurale (SANRU), mais ces interventions étaient localisées et peu coordonnées. La configuration actuelle de ces acteurs et organisations de planification familiale est relativement nouvelle. Deux entités gouvernementales – le Programme National de Santé de la Reproduction (PNSR) et le Programme National de Santé des Adolescents (PNSA) – ont le mandat d'établir des politiques et des normes pour la planification familiale et la santé sexuelle et reproductive, respectivement, mais n'ont pas suffisamment de ressources humaines et financières pour mettre en oeuvre des services de planification familiale à l'échelle nationale. De plus, aucun bailleur de fonds international n'a à lui seul les ressources pour soutenir la reconstruction d'un programme national de planification familiale qui couvrirait les 516 zones de santé de ce vaste pays. La politique sanitaire du pays est basée sur les soins de santé primaires, dont la planification familiale est l'une des composantes. Les bailleurs de fonds qui accompagnent le gouvernement de la RDC appuient les activités des soins de santé primaires dans les zones de santé du pays.

Aucun bailleur de fonds international n'a à lui seul les ressources pour soutenir la reconstruction d'un programme national de planification familiale en RDC.

Lors du retour des grands bailleurs internationaux en RDC à la moitié des années 2000, l'USAID, la Banque mondiale et le DFID ont choisi de soutenir la prestation de soins de santé dans un sous-ensemble des 516 zones de santé que compte le pays, principalement en milieu rural. Actuellement, le Projet de Santé Intégré, PROSANI, financé par l'USAID, opère dans 126 zones de santé ; le Projet d'Accès aux Soins de Santé Primaire (ASSP), subventionné par le DFID, soutient la prestation de services dans 52 zones de santé. Depuis 2013, le rôle principal du FNUAP en matière de santé reproductive et de planification familiale est d'approvisionner en commodités 63 zones de santé directement, et 150 zones de santé supplémentaires indirectement à travers au moins 6 partenaires d'exécution. La Banque mondiale soutient également la prestation des services de santé intégrés via un mécanisme de financement basé sur la performance ; son projet actuel (opérationnel depuis mai 2016) couvrira à terme 169 zones de santé additionnelles, dont 105 ne bénéficient d'aucun autre soutien externe en matière de planification familiale. Par contraste, la Fondation Bill & Melinda Gates et la Fondation David et Lucile Packard ont décidé d'investir dans la ville de Kinshasa depuis 2011 et 2013, respectivement. La carte ci-dessous illustre les

zones de santé bénéficiant d'une assistance d'un ou plusieurs partenaires extérieurs pour la planification familiale.

Dans ce contexte, le concept qui qualifie le mieux l'approche de prestation de services de la planification familiale en RDC est celui de « fragmentaire ». Les zones de santé qui bénéficient du soutien d'un ou plusieurs bailleurs de fonds internationaux ont beaucoup plus de chances de disposer d'une gamme de méthodes contraceptives modernes et de personnel qualifié que les autres ; une étude menée à Kinshasa a démontré que les formations sanitaires qui bénéficient d'un soutien externe en matière de planification familiale avaient de plus hauts niveaux d'extrants que celles qui ne bénéficiaient pas de cet appui.[6] Par ailleurs, chaque zone de santé est divisée en aires de santé. Même les grands projets de santé intégrés financés par les bailleurs de fonds ne peuvent couvrir qu'une fraction des aires de santé dans une zone de santé donnée. À la mi-2017, seuls les deux-tiers (66,4%) des 516 zones de santé que compte la RDC avaient reçu une assistance externe pour la planification familiale.[5] (Les zones de santé que la Banque mondiale projette de couvrir sont identifiées séparément dans la carte ci-dessous, car à la mi-2017, les commodités et services contraceptifs n'étaient pas encore disponibles dans toutes les zones de santé soutenues par la Banque mondiale. Une fois ce projet réalisé, le pourcentage de zones de santé recevant une assistance externe pour la planification familiale s'élèvera à 75,9%.)

Les mécanismes de prestation de services de planification familiale varient selon les provinces et les zones de santé, et sont largement influencés par le bailleur de fonds externe qui les soutient. Les provinces de Kinshasa et du Nord Kivu concentrent la plus grande offre de services de planification familiale : dans le cas de Kinshasa, parce qu'elle est la capitale du pays et représente 14% de la population nationale ; dans le cas du Nord Kivu, en raison des investissements intensifs des organisations humanitaires. Par contraste, les provinces principalement rurales n'ayant que peu ou aucun appui externe en matière de planification familiale (en rouge sur la carte) peuvent n'offrir aucun service de planification familiale, y compris dans les hôpitaux et centres de santé des grandes villes. Selon une étude menée en 2014, seules 33% des structures de santé en RDC offrent des services de planification familiale disponibles, dont seulement 20% fournissent des services de qualité selon les critères établis par cette étude.[7]

L'utilisation de la contraception en RDC se caractérise par 2 tendances relativement inhabituelles. D'abord, selon l'EDS de 2013–14, l'utilisation de méthodes traditionnelles (rythme, retrait, ou autre) est plus élevée (12,6%) que celle des méthodes modernes (7,8%) chez les femmes mariées.[3] Ensuite, le préservatif masculin est de loin la méthode la plus utilisée chez les femmes mariées utilisant la contraception moderne (43,6%), suivi de l'injectable (15,4%) et de la stérilisation féminine, la pilule et l'implant (chacune à 9,0%.).[3] Le seul autre pays d'Afrique subsaharienne affichant une utilisation de la contraception traditionnelle plus élevée que la contraception moderne est le Congo-Brazzaville (sur l'autre rive du fleuve Congo en face de Kinshasa).[8] Le préservatif masculin est la première méthode de contraception moderne dans seulement 18% des pays d'Afrique subsaharienne.[9] Plusieurs facteurs peuvent expliquer la forte dépendance aux méthodes traditionnelles en RDC : des années de troubles politiques qui ont mené à la pénurie de tous les biens matériels, une peur profondément enracinée des effets secondaires (en particulier la stérilité) des méthodes de contraception moderne, et la promotion des méthodes naturelles par les organisations confessionnelles, qui administrent plus de la moitié des formations sanitaires du pays. L'utilisation élevée du préservatif masculin pourrait être le reflet de sa disponibilité – gratuit ou vendu à très faible coût – en raison de sa distribution répandue à travers les programmes de prévention du VIH.

Le secteur privé à but lucratif (les pharmacies et les officines) constitue la principale source d'approvisionnement en méthodes de contraception pour les utilisatrices selon toutes les enquêtes récentes (EDS, Performance Monitoring and Accountability 2020 [PMA2020]), en grande partie parce que le préservatif est la méthode moderne la plus communément utilisée. Par contraste, les utilisatrices de la stérilisation féminine, de l'implant et de l'injectable se tournent vers des structures statiques : les hôpitaux ou centres de santé publics ou privés.[3] Deux grands groupes de marketing social, PSI et DKT International, fournissent la pilule, le préservatif, la contraception d'urgence, et l'injectable sous-cutané depot medroxyprogesterone acetate (DMPA-SC), connu localement sous le nom de marque Sayana Press, sur des points de vente et à un prix subventionné, principalement en milieu urbain. Les contraceptifs sont disponibles dans un nombre très limité de pharmacies dans les principales zones urbaines, et au prix fort non subventionné, mais peu d'utilisatrices ont le pouvoir d'achat suffisant pour se procurer ces méthodes auprès de ces sites de

Le concept qui qualifie le mieux l'approche de prestation de services de la planification familiale en RDC est celui de « *fragmentaire* ».

A la mi-2017, seuls les deux-tiers (66,4%) des 516 zones de santé que compte la RDC avaient reçu une assistance externe pour la planification familiale.

L'utilisation de méthodes traditionnelles est plus élevée (12,6%) que celle des méthodes modernes (7,8%) chez les femmes mariées.

ILLUSTRATION. Carte des Zones de Santé de la RDC recevant de l'aide pour la planification familiale.

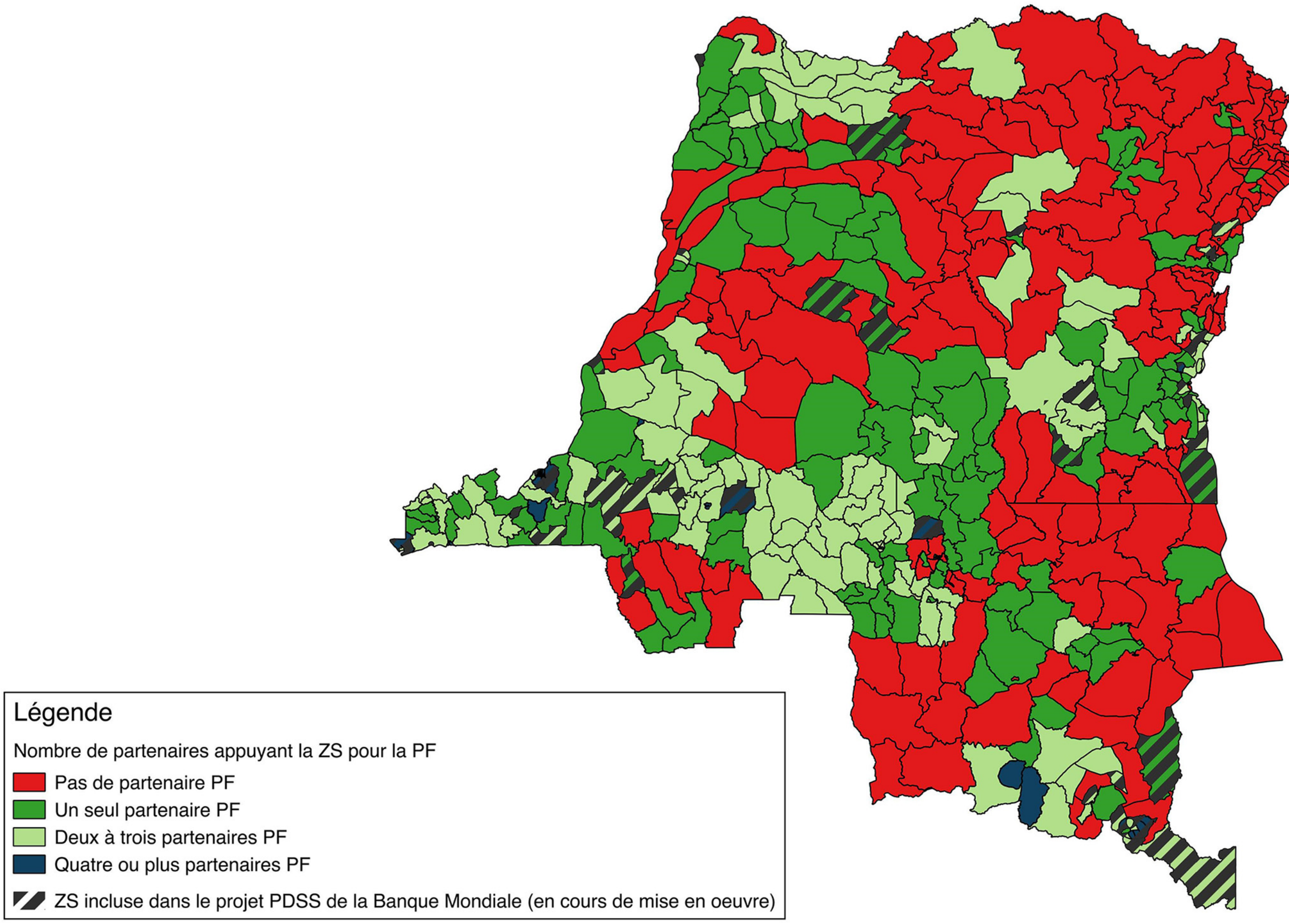

Abréviations : CHAI, Clinton Health Access Initiative ; RDC, République Démocratique du Congo ; PF, planification familiale ; ZS, zone de santé ; PDSS, Projet de Développement du Système de Santé.

Source : Clinton Health Access Initiative.[5] Carte préparée par Dr. Julie Hernandez.

prestation de santé, et les pharmacies ne réalisent que de faibles bénéfices sur ces ventes. Par ailleurs, aucun contraceptif n'est fabriqué en RDC ; ils sont tous importés, soit via des mécanismes internationaux (USAID, FNUAP et IPPF), soit achetés directement auprès des fabricants (par exemple, l'Allemagne, l'Inde, la Malaisie et la Thaïlande). « Le rayon 'contraception' est sous-développé, voire inexistant, en pharmacie en RDC », selon un spécialiste en marketing (communication personnelle, Jacques-Antoine Martin, 2018). « Les produits sont cachés derrière le comptoir, à l'exception du préservatif, et les clientes 'chuchotent' le nom du contraceptif qu'elles sont venues acheter. »

La distribution à base communautaire a pris deux formes différentes. Dans le cadre des programmes de santé rurale à grande échelle (PROSANI, ASSP), les agents de santé communautaire, connus comme « relais communautaires », fournissent la pilule et le préservatif au niveau communautaire. À Kinshasa et au Kongo Central, le projet AcQual II a établi un réseau de plus de 1000 distributeurs à base communautaire (DBC), sans formation médicale, pour fournir la pilule, le préservatif et le collier du cycle (un outil aidant les femmes à identifier et compter leurs jours féconds et non féconds en utilisant la méthode de planification familiale dite des Jours Fixes).

De 2000 à 2012, la RDC a travaillé sans objectif clair en matière de planification familiale. Le gouvernement du pays a commencé à exprimer son intérêt pour la planification familiale de manière concomitante au Sommet de Londres pour la Planification Familiale de 2012. Les membres du gouvernement et d'autres parties prenantes de la planification familiale ont canalisé cet intérêt dans l'élaboration du *Plan Stratégique National à Vision Multisectorielle pour la Planification Familiale : 2014–2020,* qui s'est fixé pour objectif d'augmenter le TPCm des femmes en âge de procréer de 6,5% (en 2013) à 19% à l'horizon 2020.[10] Cette ambition est-elle réalisable dans un pays dont le programme national ne dispose pas des ressources humaines et financières suffisantes pour assurer l'accès à la contraception à la fois dans les zones rurales et urbaines de son vaste territoire? Quelles sont les stratégies les plus prometteuses pour atteindre cet objectif?

UNE PROGRESSION ENCOURAGEANTE

De la volonté politique

Pendant la première décennie des années 2000, plusieurs organisations ont offert des services de planification familiale en RDC, en l'absence d'un leadership fort et d'une vision claire pour la planification familiale. Le premier signe d'un regain d'intérêt pour ce sujet est apparu en 2009 avec la Deuxième Conférence Nationale pour le Repositionnement de la Planification Familiale, qui s'est déroulée sous les auspices de la Première Dame. Une recommandation importante de la conférence a été d'établir un groupe de parties prenantes clés – le Comité Technique Multisectoriel Permanent (CTMP) – pour guider les initiatives de planification familiale à venir dans le pays. En raison d'un manque de ressources et de mécanismes organisationnels, ce comité a initialement bénéficié de peu de suivi.

Comme publié précédemment dans cette revue,[11] il aura fallu attendre 2012 pour que le gouvernement de la RDC commence à attribuer une plus grande importance à la question de la planification familiale. Une série d'événements positifs à partir de cette date a donné lieu à un grand soutien politique en faveur de la planification familiale émanant du Cabinet du Premier Ministre. Lors d'une conférence locale tenue en juin 2012 pour appeler à l'augmentation du financement de la planification familiale, le Ministère de la Santé s'est engagé à financer ce domaine de la santé. Participant d'un enthousiasme grandissant pour la planification familiale, le Premier Ministre a signé une lettre adressée au Ministre du Plan en 2013, lui indiquant de prioriser la planification familiale. Le deuxième document de *Stratégie de Croissance et de Réduction de la Pauvreté (DSCRP-2 2011–2015)*[12] souligne les stratégies de développement du pays, dont le besoin d'accéder aux services de santé reproductive et de planification familiale.

En 2013, le gouvernement de la RDC s'est officiellement engagé en faveur de la planification familiale lors de la Troisième Conférence Internationale sur la Planification Familiale à Addis Abeba.[13] Peu de temps après, le gouvernement a lancé son *Plan Stratégique National à Vision Multisectorielle pour la Planification Familiale (2014–2020)*. En 2014, le gouvernement a financé pour la première fois et à hauteur de 300 000 dollars l'achat de contraceptifs. En décembre 2014, il a promis une allocation supplémentaire de 2,5 millions de dollars lors d'une table ronde à la Troisième Conférence Nationale sur le Repositionnement de la Planification Familiale. En décembre 2015, le Parlement a approuvé une ligne budgétaire de 3,5 millions de dollars pour l'achat de contraceptifs dans le budget national. En 2016, le gouvernement de la RDC a déboursé 1 million de dollars pour l'achat de contraceptifs. Cette même année, la RDC était le seul pays à envoyer une délégation de trois ministres (Santé, Éducation et Plan) à la Quatrième Conférence Internationale sur la Planification Familiale à Nusa Dua, en Indonésie, où ces derniers ont présidé plusieurs panels. Par ailleurs, le Premier Ministre de la RDC a prononcé un discours via vidéo en plénière lors de cette conférence.

Le Programme National de Santé de l'Adolescent (PNSA) a également émergé comme une force importante en RDC. En 2015, le PNSA a organisé une Table Ronde sur la Santé Sexuelle et Reproductive des Adolescents et des Jeunes, qui a donné plus de visibilité et de cohérence aux partenaires travaillant dans ce domaine. L'année suivante, le PNSA a rassemblé les bailleurs de fonds et les partenaires d'exécution pour élaborer et publier le *Plan National de la Santé et du Bien-Être des Adolescents et des Jeunes (2016-2020)*, l'un des premiers de ce type en Afrique subsaharienne.

Encouragés par des signes clairs de volonté politique, le nombre de bailleurs de fonds investissant dans la planification familiale en RDC a augmenté significativement au cours des cinq dernières années.

L'investissement des bailleurs de fonds en hausse

Encouragés par des signes clairs de volonté politique, le nombre de bailleurs de fonds investissant

dans la planification familiale en RDC a augmenté significativement au cours des cinq dernières années, tout comme le montant en dollars américains de leurs investissements. Alors que plusieurs grands bailleurs (USAID, DFID, Banque mondiale) continuent de concentrer leur soutien sur des projets de services de santé intégrés à grande échelle en milieu rural, plus récemment, les bailleurs de fonds ont eu tendance à appuyer de nouvelles approches de la prestation de services, afin de tester des stratégies innovantes et de financer des programmes destinés aux jeunes et aux adolescents, plus particulièrement en milieu urbain.

On compte parmi les exemples de nouveaux investissements majeurs, deux programmes de cinq ans qui ont pour but d'étendre la couverture de la planification familiale et de réduire les écarts de financement pour l'achat des contraceptifs : le Mécanisme de financement mondial (Global Financial Facility, ou GFF) sous l'impulsion de la Banque mondiale, et l'Initiative pour la forêt de l'Afrique centrale (CAFI)/Réduire les émissions liées à la déforestation et la dégradation de la forêt (REDD+) à travers l'Agence norvégienne pour le développement international (Norad). En 2015, la RDC a été sélectionnée parmi les premiers pays cibles du GFF, initiative lancée cette même année pour promouvoir la santé reproductive, maternelle, néonatale, infantile et de l'adolescent.[15] Le cadre d'investissement du pays élaboré pour le GFF comprend la planification familiale comme une intervention prioritaire qui sera mise en oeuvre dans 14 provinces affichant parmi les plus mauvais résultats de santé reproductive, maternelle, néonatale, infantile et de l'adolescent. CAFI/REDD+ est un projet environnemental qui comprend un élément de planification familiale, reconnaissant le rôle de la croissance démographique dans la déforestation accélérée du bassin du Congo. Par ailleurs, deux donateurs anonymes ont commencé à investir dans des activités de prestation de services et de création de la demande en RDC ces dernières années. Depuis 2016, le gouvernement néerlandais a rejoint la liste des bailleurs de fonds internationaux de la planification familiale, en appuyant un programme destiné aux jeunes dans deux provinces du Kivu sous le nom *Jeunes S3* (Sécurité, Santé et Sexualité), intervenant sur la gestion de la chaîne d'approvisionnement de 2016 à 2020.

Un climat d'innovations

Dans d'autres domaines de santé publique, la RDC a souvent pris du retard en comparaison à d'autres pays de la région en termes d'innovation et d'expérimentation, en partie pour les raisons susmentionnées de ressources humaines et financières limitées. Cependant, dans le domaine de la planification familiale, la RDC s'est avérée être un pôle d'innovation. En 2012, le site internet www.familyplanning-drc.net (également disponible en français sur www.planificationfamiliale-rdc.net) a été le premier de ce type à fournir des informations sur un large éventail de sujets liés à la planification familiale en RDC. En 2013, la RDC a été le deuxième pays sélectionné pour participer au programme d'enquêtes Performance Monitoring and Accountability (PMA2020), un mécanisme innovant de collecte de données auprès de la population et des formations sanitaires : des enquêtrices résidant dans des « zones de dénombrement » recueillent des données sur des téléphones portables et les transmettent directement sur un serveur « *cloud* » pour qu'elles soient rapidement analysées.[16] En 2015, une étude pilote sur l'introduction du DMPA-SC a démontré l'acceptabilité et la faisabilité de la distribution à base communautaire de cette méthode (offerte parmi d'autres méthodes) par des étudiants en médecine et sciences infirmières.[17] La RDC mène actuellement un effort de grande envergure pour étendre l'utilisation de la plateforme DHIS 2 comme système d'information sanitaire de routine, afin d'analyser la disponibilité des contraceptifs sur l'ensemble du territoire, d'incorporer les données du système d'information géographique (SIG) pour éviter le besoin de cartographie additionnelle, et d'utiliser DHIS 2 comme système d'information complémentaire pour la gestion logistique.

En 2014, peu de programmes se focalisaient sur les problèmes de santé sexuelle et reproductive des adolescents et des jeunes. Seules 20 des 516 zones de santé offraient des services de santé sexuelle et reproductive aux adolescents et aux jeunes, de tout type que ce soit. Cependant, peu de temps après la Troisième Conférence Nationale pour le Repositionnement de la Planification Familiale en décembre 2014, le climat des programmes destinés aux jeunes a changé. Le PNSA a travaillé avec ses partenaires techniques pour développer un paquet de services de santé sexuelle et reproductive, comprenant à la fois une composante clinique et un élément de sensibilisation et services mobiles. Une Table Ronde sur la Santé Sexuelle et Reproductive des Jeunes et des Adolescents, tenue en décembre 2015, a présenté les projets émergeants, mais néanmoins grandissants dans ce domaine. En 2016, le PNSA a dirigé

l'élaboration d'un plan stratégique pour ce groupe démographique.[14] La Fondation Bill & Melinda Gates, la Fondation David et Lucile Packard, et les gouvernements de Suède et des Pays-Bas investissent dans des programmes centrés sur les adolescents et les jeunes, auxquels le FNUAP a fourni des contraceptifs. En 2017, avec le soutien du Fonds des Nations Unies pour l'enfance (UNICEF), le PNSA a cartographié tous les services de santé sexuelle et reproductive destinés aux jeunes et aux adolescents en RDC, identifiant 120 zones de santé bénéficiaires d'une assistance pour fournir ce type de services. En résumé, le soutien programmatique à la santé sexuelle et reproductive des adolescents et des jeunes en RDC, bien qu'encore naissant, a augmenté significativement au cours d'une période de 24 mois seulement.

Un mécanisme fédérateur pour la communauté de la planification familiale : Le CTMP

À l'issue de la Deuxième Conférence Nationale pour le Repositionnement de la Planification Familiale en 2009, la Comité Technique Multisectoriel Permanent (CTMP) national a été formé sur recommandation des participants, et a commencé à fonctionner en tant qu'entité coordinatrice en 2012. Ses membres comprennent plusieurs ministères (Santé, Plan, Genre), des bailleurs de fonds, et des organisations non gouvernementales (ONG) locales. Les représentants de différents bailleurs de fonds et des ONG internationales ont pu apprécier les bénéfices de ce mécanisme lors de la planification de la conférence locale sur la planification familiale de 2012. Alors que les activités de planification familiale s'accéléraient en 2013, la RDC prévoyant de s'engager publiquement à la Conférence Internationale sur la Planification Familiale d'Addis Abeba et ayant débuté l'élaboration d'un plan stratégique national, le CTMP a revêtu une toute nouvelle importance. Les réunions mensuelles du CTMP se focalisent sur un éventail de problématiques, souvent dépendantes des problèmes d'actualité (par exemple, la loi de santé reproductive, la participation aux conférences internationales). Le CTMP offre un espace de discussion permettant aux partenaires d'exécution de s'intégrer rapidement à la plus large communauté, et aux bailleurs de fonds en visite dans le pays de se réunir avec toutes les parties prenantes de la planification familiale en un même lieu. Bien que ce type de mécanisme ne soit en aucun cas unique ni spécifique à la RDC, il a donné une identité aux membres de la communauté de la planification familiale, qui peuvent désormais s'exprimer à l'unisson sur des questions clés. Le Cabinet du Premier Ministre a reconnu la valeur de ce groupe et conféré au CTMP un statut officiel par décret en mars 2015.

Une prévalence contraceptive moderne en évolution à Kinshasa

Les deux EDS menées en 2007[2] et 2013–14[3] n'ont montré qu'une faible augmentation du taux de prévalence contraceptive moderne (de 5,8% à 7,80% chez les femmes mariées en âge de procréer) au niveau national. Les enquêtes PMA2020, limitées à la ville de Kinshasa (et depuis juillet 2016, couvrant le Kongo Central), ont indiqué une augmentation du TPCm de 18,5% en 2013 à 23,4% à la mi-2017 chez les femmes en union, et de 31,0% à 39,6% chez les femmes non mariées et sexuellement actives à Kinshasa.[18,19] En 2017, les besoins non satisfaits de planification familiale allaient de 22 à 25% pour ces deux groupes de femmes, suggérant une forte demande latente de contraception moderne. En comparaison à la prévalence contraceptive de certains pays de l'Afrique subsaharienne, le TPCm de la RDC demeure faible. Cependant, l'augmentation constante de l'utilisation contraceptive, et l'adoption croissante des méthodes à longue durée d'action à Kinshasa depuis quelques années, sont pour le moins prometteuses.

Le soutien programmatique à la santé sexuelle et reproductive des adolescents et des jeunes en RDC a augmenté significativement au cours d'une période de 24 mois seulement.

DES DEFIS IMPRESSIONNANTS

Les systèmes de santé publics globalement faibles dans de nombreux pays d'Afrique subsaharienne ont déjà fait couler beaucoup d'encre : une infrastructure peu fiable, un manque d'équipement et de produits de base, de bas salaires, une formation et supervision du personnel insuffisante, de faibles systèmes d'information, des ruptures de stocks fréquentes des médicaments, et un mauvais accueil des clientes par les prestataires souvent irrespectueux ou culturellement peu sensibles aux problèmes d'autrui.[20,21] À cette liste nous ajoutons les défis spécifiques liés à la situation politique, ainsi qu'à l'offre et la demande contraceptive en RDC.

Le problème de l'instabilité politique

Le président actuel Joseph Kabila est entré au pouvoir en 2001 (après le meurtre de son père, le président Laurent-Désiré Kabila). Ses deux mandats constitutionnels à la tête du pays se sont

terminés en décembre 2016. L'absence d'élections en novembre 2016 a provoqué des tensions politiques et des grèves intermittentes, qui ont fait la une des journaux du monde entier.

En février 2018, de nouvelles élections ont été annoncées et fixées au 23 décembre 2018, le président entrant devant prendre ses fonctions en janvier 2019. Cependant, l'opposition continue de revendiquer le départ du président Kabila dès maintenant, plutôt que d'attendre les élections. Ainsi, l'annonce des prochaines élections n'a pas encore conduit à la stabilité politique du pays. La plupart des observateurs internationaux ont adopté une attitude attentiste.

Les bailleurs de fonds actuels et potentiels suivent de près la situation politique de la RDC, au fur et à mesure qu'ils définissent leurs prochains investissements dans la planification familiale et d'autres secteurs. Le défi est de maintenir la confiance des bailleurs de fonds, laquelle dépend d'un retour à la stabilité politique, ce qui devrait se produire à l'issue des prochaines élections nationales et à condition de leur bon déroulement. La plupart des bailleurs de fonds de la planification familiale demeurent fortement engagés et solidaires du travail déjà mis en oeuvre, mais restent vigilants face à l'instabilité politique du pays.

Les défis logistiques et de gestion de la prestation de services

La RDC est le plus vaste territoire de l'Afrique subsaharienne, cependant son infrastructure routière est extrêmement fragile. Aucune autoroute ni aucun réseau ferroviaire ne relie le pays d'Est en Ouest ; les 2794 kilomètres de chaussée goudronnée du pays sont des routes à deux voies. Plusieurs compagnies aériennes nationales desservent les principales villes du pays, mais leur bilan de sécurité est mauvais. (Les agences internationales dépendent largement du Service aérien d'aide humanitaire des Nations Unies pour les vols nationaux.) Moins de 7% de la population a accès à Internet,[22] bien que la pénétration de la téléphonie mobile par carte SIM ait atteint 39,5%.[23]

En plus des défis logistiques, les failles générales du système de santé public fragilisent la prestation des services de planification familiale : les ressources humaines et financières inadéquates pour former et superviser de manière appropriée les prestataires de santé, le manque d'équipement et de matériel nécessaires à la prestation des services, la détérioration des infrastructures physiques, dont certaines datent de l'époque coloniale (avant 1960), sont autant de difficultés auxquelles se heurtent les acteurs de la planification familiale. Les systèmes de gestion sont également insuffisants, donnant lieu à une situation où les prestataires de santé fixent les prix de leurs services selon leur bon-vouloir, plutôt qu'en fonction de prix standard établis. Les contraceptifs distribués gratuitement dans le secteur public sont souvent acheminés de manière irrégulière vers le secteur privé, où ils sont revendus lucrativement par des personnes dont les connaissances sur leur bonne utilisation peuvent être limitées.

Et comme si cela ne suffisait pas, depuis la fin 2016, des troubles sociaux ont éclaté par endroits dans le pays, exposant le personnel qui travaille ou approvisionne les formations sanitaires dans ces régions à un risque sécuritaire.

La sécurité contraceptive et la gestion de la chaîne d'approvisionnement

La sécurité contraceptive (disposer des bons contraceptifs, au bon endroit, au bon moment et au bon prix) requiert une gestion avisée et des ressources financières suffisantes pour effectuer une série de tâches complexes : quantifier les besoins contraceptifs d'une population donnée, estimer les coûts de l'achat et du transport des commodités, identifier les mécanismes d'achat, émettre des bons de commande, obtenir l'exemption des droits de douane, assurer la livraison aux dépôts centraux ou directement à ceux régionaux, assurer le transport vers les points de distribution où les clients pourront obtenir des produits de contraception, suivre le flux continu des commodités, assurer la comptabilité de toutes les dépenses, et financer enfin l'ensemble de ce processus.

De nombreux facteurs constituent des défis quant à la sécurité contraceptive de la RDC. Différents bailleurs de fonds et partenaires d'exécution de la planification familiale gèrent l'approvisionnement et la distribution des contraceptifs via des circuits parallèles au système national. Idéalement, la chaîne d'approvisionnement des médicaments essentiels du pays devrait intégrer les contraceptifs pour améliorer l'efficacité du système et réduire les coûts de transaction. Bien que des discussions soient en cours sur ce sujet, l'achat et la distribution des contraceptifs ont actuellement lieu en dehors de la chaîne d'approvisionnement des médicaments essentiels du gouvernement.

En ce qui concerne l'achat de produits contraceptifs, le gouvernement y a contribué en

apportant ses propres fonds, mais n'est en mesure de ne couvrir qu'une fraction des coûts entraînés par une population de la taille de la RDC. Les bailleurs de fonds ne peuvent pas combler cet écart. La logistique contraceptive, qui comprend l'achat, le transport, la distribution et le stockage, constitue un défi de taille, en particulier pour assurer l'acheminement vers le « dernier kilomètre » (le client).

Le poids des normes socioculturelles qui favorisent les familles nombreuses

La demande de services de planification familiale découle du désir d'éviter une grossesse sur le court terme, et d'espacer ou limiter le nombre de naissances sur le long terme. Toutes choses étant égales par ailleurs, les sociétés qui favorisent les familles nombreuses auront moins de demande de contraception, bien que ces normes évoluent au fil du temps et en réponse aux changements macroéconomiques (par exemple, l'amélioration des opportunités éducatives des femmes, l'augmentation de l'urbanisation). En RDC, l'indice synthétique de fécondité est de 6,6 enfants par femme en moyenne (5,4 en milieu urbain, 7,3 en milieu rural).[3] Comme le fait remarquer Romaniuk, l'incapacité d'un couple à subvenir aux besoins de leurs enfants, et les opportunités limitées des femmes d'intégrer la population active, n'ont pas d'effet dissuasif sur la taille des familles.[24]

La recherche qualitative récente met en lumière de nombreux facteurs qui favorisent la persistance d'une forte fécondité.[25,26] Avoir beaucoup d'enfants est un signe de statut social autant pour les hommes que pour les femmes. De plus, la loi congolaise fait des femmes des subordonnées de leurs époux dès le jour de leur mariage, ce qui leur confère moins de pouvoir dans leur relation. La famille de l'époux peut estimer que l'épouse de ce dernier lui doit beaucoup d'enfants en échange de la dote payée pour le mariage. Si elle ne met pas au monde ces enfants, la famille de l'époux pourra encourager ce dernier à épouser une autre femme. Dans la mesure où les parents dépendent de leurs enfants en vieillissant, avoir beaucoup d'enfants est une manière de s'assurer que quelqu'un subviendra à leurs besoins plus tard. Enfin, les enfants sont une importante source de main d'oeuvre pour les familles, particulièrement en milieu rural.

Les normes culturelles changent en réponse aux pressions économiques subites par les familles nombreuses, particulièrement en milieu urbain. Toutefois, même les femmes des quintiles de bien-être économique les plus élevés dans les zones urbaines de la RDC souhaitent avoir 4,9 enfants en moyenne ; et un plus haut niveau d'études en RDC n'a un effet que minimal sur le désir d'avoir de nombreux enfants.[2]

Les frais d'accès aux services

Le PIB par habitant de la RDC est de $405.[27] Le pays est classé 176ème sur 188 selon l'Indice de Développement Humain.[28] Avec 77,1% de sa population vivant avec moins de 1,90 dollar par jour, la RDC figure parmi les pays les plus pauvres du monde. En RDC, 70% du système de santé public est financé par les frais d'accès aux services, c'est-à-dire payés par les ménages, et seulement 13% est couvert par le gouvernement, et 14% par les bailleurs de fonds extérieurs.[29] Ce mode de paiement direct par les ménages constitue un obstacle financier conséquent à l'accès aux soins pour les couches les plus pauvres de la population.

Dans ce contexte d'extrême pauvreté, le coût des contraceptifs peut devenir une barrière infranchissable. Les programmes desservant les zones rurales (ex : PROSANI, ASSP) offrent des contraceptifs gratuitement. Cependant, les pharmacies agréées dans les zones urbaines affichent des prix correspondant à ceux du marché ou aux prix subventionnés (lorsqu'elles reçoivent des commodités de programmes de développement). Les hôpitaux et les centres de santé en milieu urbain fournissent souvent des contraceptifs subventionnés par les principaux bailleurs de fonds ; cependant, même lorsque le produit est moins cher, voire gratuit, les formations sanitaires peuvent faire payer aux client(e)s des frais de consultation et d'utilisation de produits nécessaires à l'administration des méthodes contraceptives (ex : seringue, coton et alcool pour les injections ou l'insertion de l'implant). Les programmes de marketing social, dont PSI et DKT International, vendent une gamme de contraceptifs à des prix subventionnés. De plus, PSI, à travers l'Association de Santé Familiale (ASF), a instauré un système dit « *payant et gratuité* », à travers lequel des contraceptifs sont distribués gratuitement une fois par mois, les rendant plus accessibles à ceux qui ne pourraient autrement pas les payer.

Selon FPwatch, une étude sur les points de distribution menée en 2015 à Kinshasa et dans la province du Katanga, le prix médian par Année Protection Couple (APC) à Kinshasa était de 1,75 dollar américain (et légèrement plus élevé

dans le Katanga).[30] Le coût par APC était de 4,95 dollars américains pour l'implant et de 0,55 dollar américain pour le dispositif intra-utérin. Certaines formations sanitaires à Kinshasa font payer le très populaire implant entre 10 et 20 dollars l'unité, et les clientes doivent débourser le montant complet de la méthode le jour de la consultation, même si le coût annuel sur plusieurs années de protection est bien moindre.

Les prix des contraceptifs ne sont pas standardisés, ce qui aggrave l'incapacité des clients à en assumer le coût. Le PNSR a établi une liste de prix pour tous les contraceptifs vendus dans les structures publiques, mais ces derniers ne sont pas en vigueur. De plus, les prix sont rarement affichés. En 2017, seuls 23% des sites de prestation de santé qui facturaient leurs services de planification familiale à Kinshasa affichaient les prix de leurs contraceptifs.[19]

PERSPECTIVES D'AMÉLIORATION DE LA COUVERTURE NATIONALE DE LA PLANIFICATION FAMILIALE

Le Plan National Stratégique à Vision Multisectorielle pour la Planification Familiale (2014–2020) appelle à une augmentation de la prévalence contraceptive moderne à 19% à l'horizon 2020. Bien que le PNSR et le PNSA remplissent un rôle normatif et coordonnent des activités spécifiques, aucun de ces programmes n'a le soutien financier suffisant, ni du gouvernement ni des bailleurs de fonds, pour assurer la prestation de services de planification familiale au niveau national. Rien ne suggère aujourd'hui que le gouvernement ou les bailleurs de fonds pourraient apporter les ressources suffisantes pour soutenir un programme national qui aspirerait à donner accès à la contraception à tous et partout sur le territoire de cette vaste nation.

Plusieurs initiatives clés en cours pourraient, si elles s'avèrent efficaces, contribuer significativement à améliorer l'accès à la contraception. Elles s'alignent sur les 6 piliers du cadre de travail pour le renforcement des systèmes de santé de l'Organisation mondiale de la santé (OMS) : gouvernance et leadership, prestation de services, personnel, commodités, information et financement.[31] Les initiatives présentées ci-dessous illustrent (de manière non exhaustive) le travail en cours dans le pays soutenant chacun de ces six piliers.

Gouvernance et leadership : Transformer le réseau des CTMP provinciaux en une alliance nationale pour la planification familiale

Étant donnée l'efficacité du CTMP national en tant que mécanisme de coordination apportant une cohérence à la communauté de la planification familiale, des efforts ont commencé à voir le jour en 2016 pour établir des CTMP au niveau provincial. Sur les 26 provinces que compte la RDC, 12 ont actuellement un CTMP provincial (Kinshasa, Bas Uele, Kasai Central, Katanga, Kongo Central, Lualaba, Nord Kivu, Sankuru, Sud Kivu, Tshopo, Ituri et Lomani). Le Partenariat de Ouagadougou, un groupe de 9 pays de l'Afrique de l'Ouest francophone ayant joint leurs forces pour atteindre un même objectif de planification familiale, sert d'exemple pour ce partenariat en RDC.[32]

Pour établir un CTMP provincial, la présence d'une ONG internationale fournissant des services de planification familiale dans la province est essentielle, tout comme sa disposition à remplir un rôle de coordination et à soutenir financièrement l'organisation des réunions du CTMP. Le PNSR participe pleinement à cette initiative. Tel que structurés actuellement, les CTMP provinciaux sont composés d'acteurs gouvernementaux, dont les représentants provinciaux du PNSR, de la Division Provinciale de la Santé (DPS), d'au moins 3 ministères (Santé, Plan et Genre), et dans certains cas, des ministères de l'Environnement et des Affaires Sociales, ainsi que des ONG internationales et locales.

En l'absence d'un programme national capable de fournir des ressources financières aux opérations de planification familiale dans les provinces, le CTMP constitue un groupe d'acteurs bénévoles et engagés pour faire avancer la planification familiale au niveau provincial. Les CTMP provinciaux ne sont pas des entités opérationnelles ; ils n'ont pas de budget pour mener des activités programmées. Cependant, ces groupes de parties prenantes provinciales pourront superviser l'évolution de la prestation de services de planification familiale de multiples manières. Ils sont déjà impliqués dans la quantification des besoins contraceptifs dans leurs provinces respectives. Ils auront prochainement accès aux statistiques de services, qui s'améliorent rapidement, grâce à l'adoption du système DHIS 2 en RDC. Ces données permettront aux provinces de suivre le pourcentage de zones de santé (et des aires de santé qu'elles regroupent) offrant des services de planification familiale. Ce type de

programmation fondée sur des données prouvées est susceptible d'encourager les bailleurs de fonds existants à combler certains manques en matière de programmation, et d'attirer des nouveaux investissements. L'amélioration des compétences de plaidoyer du personnel provincial aidera à sécuriser ces gains.

Trois activités clés en 2017 ont façonné cette nouvelle initiative. En mars, les membres des CTMP provinciaux se sont réunis à Kinshasa pour la première fois, témoignant d'une solidarité envers ce type d'approche coordonnées pour promouvoir la planification familiale en RDC. En septembre, PATH a organisé une formation de 3 jours, à laquelle ont assisté 2 personnes par province, afin de renforcer leurs compétences et techniques de facilitation de sessions stratégiques fondées sur les outils de plaidoyer SMART d'Advance Family Planning (AFP).[33] En octobre, une série d'ateliers de trois semaines ont été organisés pour couvrir les 12 CTMP provinciaux, auxquels ont participé 4 membres par province pour suivre une formation sur les fondamentaux des programmes de planification familiale, le fonctionnement autonome du CTMP provincial, l'amélioration de la quantité et qualité de la couverture de la planification familiale, et le suivi des progrès en utilisant les statistiques de services.

Bien que les CTMP provinciaux opèrent de manière relativement autonome, le CTMP national continuera de remplir un rôle important en tant que coordinateur et catalyseur. Il organisera des réunions annuelles durant lesquelles chaque province présentera ses accomplissements et ses domaines à améliorer ; il coordonnera les formations et ateliers de renforcement de compétences au niveau provincial ; il créera une plateforme de communication via un nouveau site internet : www.ctmp-pf.org

La prestation de services : Les initiatives visant à améliorer l'offre et la demande

La structure de la prestation de services en RDC ressemble à celle de la plupart des pays en voie de développement. Les services sont disponibles via des formations sanitaires statiques (par exemple, les hôpitaux, les centres de santé, les postes sanitaires), les pharmacies, et les agents de distribution communautaire. Les services sont financés à travers le secteur public, le secteur privé à but lucratif, et le secteur privé à but non lucratif (les ONG), dont les organisations confessionnelles. Des structures « hybrides » existent également : elles appartiennent au gouvernement qui en paie les employés, mais c'est une ONG qui est responsable de former le personnel et de fournir les commodités contraceptives.[6] En plus des pharmacies agréées, de nombreuses pharmacies informelles (officines) fournissent également une ou plusieurs méthodes de contraception, comme le préservatif.

Trois initiatives peuvent être citées en exemple, ayant été conçues pour augmenter l'offre, améliorer la qualité, et créer de la demande. Sans toutefois être uniques ni propres à la RDC, ni innovantes sur la scène internationale, ces initiatives représentent des tentatives prometteuses pour améliorer la prestation de services en RDC.

Les programmes de marketing social

Autant PSI que DKT International mettent en oeuvre des programmes de marketing social en RDC, ce qui consiste à promouvoir la vente de contraceptifs subventionnés via de multiples circuits, à la fois traditionnels (pharmacies et officines) et non traditionnels (bars, hôtels, stations services).

Le travail de DKT International illustre certaines innovations actuellement à l'oeuvre en RDC. La Brigade Intervention de Plaisir Protégé opère sur le modèle d'une brigade de sécurité dont les « agents » circulent à l'arrière d'une camionnette reconnaissable par son nom de marque clairement affiché. La camionnette s'arrête devant un bar, et les agents (de jeunes femmes et hommes attrayants) se précipitent à l'intérieur de l'établissement et se mélangent aux clients, afin de promouvoir la vente de préservatifs comme une « arme ». En plus d'assurer la notoriété de la marque et de déstigmatiser la contraception, cette stratégie a démontré la volonté des clients de payer pour ces produits. Une autre stratégie repose sur un réseau de « *Bees* » (abeilles) : des infirmières dûment formées, principalement des femmes, se rendent dans différentes communautés pour fournir la pilule, le préservatif, et le DMPA-SC, ciblant les femmes à faibles revenus pour qui se rendre dans une clinique représente une barrière financière. Les clientes qui choisissent le DMPA-SC reçoivent un « jeton » avec la date de leur prochaine injection, et peuvent choisir de recevoir une notification par SMS. Si une cliente souhaite obtenir une méthode clinique, les infirmières leur donnent un « jeton de référence » vers une formation sanitaire disposant de personnel qualifié. Les infirmières perçoivent 50% des ventes du produit, ce qui correspond à 0,50 dollars par injection du DMPA-SC.

Sur une période de 6 mois en 2017, elles ont vendu 90 000 unités du DMPA-SC.

DKT International est également connu comme une organisation favorable aux jeunes en RDC, et s'adresse aux jeunes de 15 à 24 ans en particulier. Cette ONG utilise une approche centrée sur l'humain pour créer des programmes de santé sexuelle et reproductive destinés aux jeunes de cette tranche d'âges, appelés *Batela Lobi Na Yo* (« Protège ton avenir ») afin d'augmenter l'utilisation de produits et services de planification familiale de qualité chez les jeunes femmes en RDC.

Renforcer les services de planification familiale à travers l'armée

Des membres de l'armée à Kinshasa ont approché le PNSR et d'autres acteurs pour obtenir leur soutien afin d'introduire et de renforcer les services de planification familiale dans les formations sanitaires administrées par l'armée. Ils ont également sollicité l'application d'une enquête auprès de la population pour mieux comprendre en quoi l'utilisation de la contraception chez les populations vivant dans des camps militaires était différente de celle de la population civile. L'étude a montré qu'en 2016, le TPCm des femmes en union vivant dans les camps militaires était bien plus faible (16,0%) que celui de la population totale (23,4%).[34] Dans la mesure où les camps militaires sont souvent considérés comme des « lieux difficiles », les programmes précédents les ont évités. Aujourd'hui, ils sont partenaires des programmes, forment le personnel des structures sanitaires statiques, établissent des points de distribution à base communautaire, et affichent des membres de l'armée sur des panneaux publicitaires promouvant la planification familiale à Kinshasa, ainsi que dans la province adjacente du Kongo Central. Étant donnée la mobilité du personnel de l'armée en RDC, il est probable que des changements de comportements au sein de cette population génèrent des bénéfices dans d'autres parties du pays.

Créer la demande de services de planification familiale

Avant 2015, la création de la demande consistait principalement en du marketing social dans les villes principales du pays, et la distribution périodique de documentation imprimée en quantité limitée. Depuis 2015, plusieurs organisations travaillant dans la communication pour le changement des comportements (dont le PNSR), avec le soutien du Center for Communication Programs (Centre pour les Programmes de Communication) de l'Université de Johns Hopkins, ont formé une équipe centrale qui a produit plusieurs contenus : un logo unique et amélioré devant être utilisé par toutes les organisations de planification familiale ; une brochure s'adressant aux publics avec de faibles niveaux d'alphabétisation et portant sur la gamme de méthodes contraceptives disponibles ; et des panneaux publicitaires encourageant la communication intraconjugale sur la planification familiale. Ce groupe a travaillé avec des chaînes de télévision locales pour faire de la planification familiale un thème central d'une émission sur les jeunes mariés, et a diffusé des messages sur la planification familiale via des spots publicitaires dans une émission de radio populaire dédiée aux problèmes de santé. Une ligne téléphonique (hotline) offre aux jeunes, et aux autres usagers, un moyen confidentiel d'obtenir des informations fiables sur la contraception. Le groupe a également développé et testé une méthodologie pour les activités à base communautaire. Ces programmes ont pour but d'influencer autant les comportements individuels que les normes sociales au niveau communautaire.

Le personnel : Institutionnaliser la planification familiale via un réseau national d'Écoles de sciences infirmières

En 2015, une recherche pilote sur l'acceptabilité et la faisabilité de confier la distribution des contraceptifs, dont le DMPA-SC, au niveau communautaire à des étudiants en médecine et sciences infirmières, a généré de nouvelles connaissances sur le potentiel de cette catégorie de travailleurs comme prestataires de services de planification familiale en RDC.[32] Alors que cette recherche pilote s'était donnée pour objectif de tester l'acceptabilité du DMPA-SC à travers sa distribution au niveau communautaire, sa plus grande « découverte » repose sur le potentiel que représentent ces professionnels de la santé en formation pour étendre l'accès à la contraception. Ces étudiants sont dynamiques, instruits, désireux d'acquérir de l'expérience au niveau communautaire, et jeunes (pouvant donc être approchés plus facilement par leurs pairs cherchant à obtenir des contraceptifs). De plus, ils sont les leaders du système de santé de demain en RDC.

La 6ème Direction (D6) du Ministère de la Santé supervise les 477 écoles de sciences infirmières et de sages femmes du pays. (Les écoles de médecine

opèrent de manière plus autonome sous une Direction de tutelle différente). En 2016, des efforts ont vu le jour dans le but d'institutionnaliser la planification familiale dans les écoles de sciences infirmières. En particulier, ces efforts visaient à établir un curriculum sur la planification familiale que tous les étudiants en sciences infirmières devraient suivre comme premier module de troisième année ; former les professeurs de ces écoles à enseigner cette matière ; et incorporer un stage au niveau communautaire dans le cursus des étudiants, qui fourniraient ainsi cinq méthodes contraceptives (Implanon NXT, DMPA-SC, la pilule, le préservatif et le collier du cycle) au niveau communautaire. En raison de la popularité croissante de l'implant en RDC, l'utilisation de cette catégorie de prestataires pour fournir Implanon NXT en milieu non clinique permettrait d'améliorer l'efficacité potentielle de cette stratégie.

À ce jour, la D6, le PNSR, et d'autres parties prenantes ont instauré ce programme dans 11 écoles de sciences infirmières à Kinshasa, et 6 écoles de sciences infirmières dans la province adjacente du Kongo Central. Les étudiants y opèrent en tant que distributeurs à base communautaire. Le Système National d'Information Sanitaire (SNIS) est actuellement en train de modifier ses formulaires de collecte de données sur la planification familiale pour refléter la contribution de cette nouvelle catégorie du personnel.

Ce mécanisme innovant représente une première étape vers l'expansion et le renforcement de la prestation des services de planification familiale en RDC. Il ne représente en aucun cas une solution complète à ce défi d'envergure. Cependant, il a émergé comme l'un des moyens potentiellement les plus efficaces pour relancer l'offre de services, particulièrement pour l'étendre aux provinces au-delà de Kinshasa.

Les commodités : Assurer la sécurité contraceptive à travers la gestion améliorée de la chaîne d'approvisionnement

On peut difficilement exagérer l'envergure du défi que représente la sécurité contraceptive dans un pays dont le territoire est immense, les systèmes de transport fragiles, le financement pour l'achat de contraceptifs largement insuffisant, et où un système d'achat et de distribution unifié fait défaut. La RDC ne peut se vanter d'avoir « innové » dans ce domaine. Le pays doit plutôt se focaliser sur l'établissement des éléments de base d'un système logistique fonctionnel pour l'acquisition de contraceptifs, ce qui constitue en soi un objectif ambitieux, bien que fondamental, pour réussir à augmenter le TPCm. Ce défi logistique de taille a mené certains observateurs à suggérer l'idée que la RDC pourrait servir de banc d'essai pour l'utilisation de drones comme moyen de livraison des commodités contraceptives.

On compte parmi les étapes positives vers l'amélioration de la sécurité contraceptive :

- L'établissement d'une équipe multi-agences chargée de la logistique contraceptive, composée du gouvernement, des ONG internationales et des bailleurs de fonds ;
- La quantification des besoins en contraceptifs du pays, par province et pour la période 2016–2020 ;
- L'estimation du coût de l'approvisionnement en contraceptifs et de la prestation de services de planification familiale, ainsi que l'écart financier existant pour satisfaire ces besoins ;
- L'établissement d'un système d'information en gestion logistique pour les ruptures de stocks de contraceptifs au niveau des zones de santé, avec des mécanismes permettant de déclencher le réapprovisionnement ;
- Le plaidoyer auprès des bailleurs de fonds de la santé pour allouer un budget aux contraceptifs.

La planification familiale a été institutionnalisée dans 11 écoles de sciences infirmières à Kinshasa, et dans la province adjacente de Kongo Central, dont les étudiants opèrent en tant que distributeurs à base communautaire.

On compte parmi les activités clés en cours, ou en phase de planification, qui devraient contribuer à améliorer l'efficacité de la logistique contraceptive :

- La mise en oeuvre d'un plan national stratégique pour la chaîne d'approvisionnement, afin d'améliorer l'alignement et l'efficacité du gouvernement, des bailleurs de fonds et des partenaires (récemment signé par le Ministère de la Santé) ;
- Le développement d'une plus grande cohésion et coordination entre les membres de l'équipe centrale inter-agences chargée de la logistique contraceptive ;
- Le développement d'un système d'information fiable, intégral et transparent sur l'achat et la distribution des contraceptifs importés dans le pays et mis à disposition du secteur public, privé et à but non-lucratif ;
- Le maintien d'une forte initiative de plaidoyer pour mobiliser des fonds auprès de bailleurs de fonds internationaux afin de combler le manque de financements des contraceptifs.

En 2016, une initiative a commencé à évaluer la possibilité d'incorporer des contraceptifs au système d'approvisionnement et de distribution plus large des commodités essentielles, soutenu par le gouvernement. Au coeur de ce système repose FEDECAME, une organisation quasi-gouvernementale, responsable de l'achat des commodités de santé en RDC. FEDECAME est un réseau composé d'une unité de coordination chargée des achats et de la chaîne d'approvisionnement, basée à Kinshasa, et reliée à 19 centrales de distribution régionale (CDR) des médicaments dans le pays ; son mandat est d'assurer l'approvisionnement en commodités sanitaires de tout le système de santé, jusqu'au niveau communautaire. FEDECAME est parvenue à approvisionner le pays en nombreux médicaments essentiels, mais elle manque d'expérience dans l'approvisionnement des commodités servant les programmes de planification familiale, VIH/SIDA et tuberculose, entre autres (en raison d'arrangements historiques selon lesquels les bailleurs verticaux tels que PEPFAR, le Fonds mondial, ou encore l'Initiative Présidentielle contre le Paludisme, assurent l'approvisionnement de commodités spécifiques).

Cependant, cette dernière option devrait-elle continuer d'être l'objectif à poursuivre sur le long terme? Une stratégie de long terme (par exemple, 10 ans) raisonnable pourrait peut-être se reposer sur une chaîne d'approvisionnement appartenant à l'Etat et intégrée au secteur public, avec des étapes intermédiaires importantes à franchir, tout en recevant une aide significative des bailleurs de fonds.

L'information : Renforcer les systèmes d'information pour surveiller le progrès

Les progrès significatifs dans ce domaine devraient être mesurés non pas à l'aune des pays de l'Afrique subsaharienne qui affichent une prévalence contraceptive élevée et dont les programmes sont arrivés à maturité, mais plutôt de ceux qui ont encore beaucoup de retard à rattraper. Trois types d'informations sont utiles pour suivre les activités en cours et faire progresser les programmes de planification familiale : le système d'information sanitaire de routine, les enquêtes auprès de la population et des formations sanitaires, et les études spéciales.

Les systèmes d'information sanitaire de routine

Le Système National d'Information Sanitaire (SNIS) a fait des progrès remarquables dans la collecte de statistiques de services, y compris dans le domaine de la planification familiale. Depuis 2013, avec le soutien solide du projet ASSP, financé par le DFID, la 5ème Direction (D5) du Ministère de la Santé a commencé à installer la plateforme DHIS 2 dans les 11 provinces que comptait alors le pays. À la fin 2017, le système rapportait des données des 26 provinces que compte le pays aujourd'hui, avec 95% des zones de santé rapportant avoir des activités de la planification familiale. (Chaque zone de santé est subdivisée en aire de santé, qui inclut une ou plusieurs formations sanitaires faisant remonter leurs données via ce système). Bien que 95% des zones de santé rapportent des statistiques de services dans le domaine de la planification familiale, c'est le cas de seulement 71% des aires de santé, et à peine 40% des 16 465 formations sanitaires déclarent avoir un certain niveau d'activités de planification familiale. La D5 a travaillé avec le CTMP national pour examiner les données de planification familiale collectées par le SNIS. Ce système collecte les données nécessaires au calcul de l'Année Protection Couple (APC) (un indicateur courant des extrants de la planification familiale). Les membres de CTMP provinciaux sont désormais formés pour accéder à ces données directement, et les interpréter afin de surveiller les progrès atteints au niveau provincial. Par ailleurs, le système peut être utilisé pour identifier les zones de santé (et en leur sein, les aires de santé) qui n'ont pas de services de planification familiale fonctionnels. Des efforts sont à l'oeuvre pour obtenir des coordonnées GPS de toutes les formations sanitaires du système, ce qui permettra des analyses additionnelles de l'accès à la contraception.

Les enquêtes auprès de la population et des formations sanitaires

Les enquêtes représentatives auprès de la population offrent un aperçu des dynamiques de l'utilisation contraceptive sur une période donnée, dont : la prévalence contraceptive moderne, les besoins non satisfaits, la distribution des méthodes, et les déterminants de l'utilisation contraceptive, entre autres facteurs. Les enquêtes auprès des formations sanitaires décrivent l'environnement de l'offre de la planification familiale dans une aire géographique donnée. La RDC est le deuxième pays (sur 11) à avoir mené l'enquête PMA2020,[16] qui a déjà conduit 6 vagues de collecte de données à Kinshasa et 3 au Kongo Central. Les résultats de cette enquête ont tracé

une hausse de 8 points de pourcentage du TPCm, et une augmentation constante de l'utilisation de l'implant à Kinshasa sur une période de cinq ans. Une deuxième étude sur les formations sanitaires, FPwatch, menée à Kinshasa et au Katanga en 2015, a apporté des informations approfondies sur l'environnement de l'offre dans ces deux provinces. La prochaine EDS, prévue pour 2019 ou 2020, génèrera des indicateurs de progrès pour d'autres provinces et à l'échelle nationale.

Les études spéciales

Une série de recherches pilotes menées par l'Université de Tulane en collaboration avec les partenaires locaux a testé l'acceptabilité et la faisabilité de nouvelles stratégies pour augmenter l'accès à la contraception au niveau communautaire. Le premier pilote, mené en 2015, a testé l'utilisation d'étudiants en médecine et sciences infirmières pour distribuer le DMPA-SC (avec la pilule, le préservatif et le collier du cycle).[35,36] Les résultats positifs de cette étude ont mené à trois pilotes additionnels à Kinshasa. Les deux premiers se focalisent sur l'acceptabilité et la faisabilité d'utiliser la même catégorie d'étudiants pour fournir Implanon NXT et pour enseigner aux femmes de la communauté à utiliser le DMPA-SC via auto-injection. Le troisième, mis en oeuvre dans la province rurale de Lualaba, forme des relais communautaires (des agents de santé communautaire sans formation médicale) pour leur permettre d'injecter le DMPA-SC au niveau communautaire. Par ailleurs, des études utilisant des groupes focaux et des clientes mystères ont souligné des attitudes généralement favorables envers la contraception d'urgence, et la volonté des pharmacies officielles de fournir la contraception d'urgence aux jeunes femmes.[37,38] La conduite d'études spéciales pour tester continuellement de nouvelles approches et idées contribue au dynamisme de l'environnement de la planification familiale.

Le financement : Développer les flux financiers pour couvrir un programme largement sous-financé

Le financement demeure un défi majeur dans un pays aux ressources limitées et dont un très faible pourcentage du budget national est alloué à la santé. Les programmes de planification familiale en RDC sont toujours extrêmement dépendants des bailleurs de fonds, en dépit de la contribution politiquement importante, mais néanmoins modeste, du gouvernement de la RDC à l'achat des contraceptifs. La hausse de l'investissement des bailleurs de fonds observée dans les 5 dernières années ne semble pas prête de ralentir.

Au niveau international, le GFF est positionné pour mettre à profit le Fonds de la Banque mondiale pour les pays les plus pauvres en soutenant la planification familiale comme une priorité du continuum de santé reproductive, maternelle, néonatale, infantile, de l'adolescent, et de nutrition. Au niveau national, plusieurs CTMP provinciaux — Lualaba, Nord Kivu et Sud Kivu — ont créé avec succès une ligne budgétaire pour l'achat des contraceptifs dans le budget de leurs provinces.

Des efforts visant à impliquer le secteur privé pourraient contribuer à financer davantage l'offre des services de planification familiale, par exemple, dans le secteur minier. Toutefois, si la RDC aspire à la couverture universelle de la planification familiale à l'horizon 2030, il reste encore beaucoup à faire pour atteindre cet objectif ambitieux.

CONCLUSION

Certains ont interrogé les normes qui favorisent la forte fécondité en RDC en se demandant si elles sont susceptibles de changer.[26] Avant 2012, le gouvernement n'attribuait que peu d'importance à la planification familiale, et les bailleurs de fonds étaient réticents à l'idée d'investir dans la planification familiale dans un pays marqué par des défis impressionnants. Cet article apporte des connaissances approfondies sur ces défis, tout en exposant le net progrès accompli depuis 2012 en matière de soutien gouvernemental et de programmation des activités de planification familiale en RDC. Il présente des initiatives prometteuses qui devraient contribuer à l'objectif d'atteindre un taux de prévalence contraceptive moderne de 19% à l'horizon 2020, en accord avec les piliers du renforcement des systèmes de santé. Le défi que représente l'approvisionnement des commodités contraceptives en quantités suffisantes pour répondre à la demande croissante de contraception est de taille, tout comme l'est l'amélioration de la gestion de la chaîne d'approvisionnement pour assurer la livraison des services « au dernier kilomètre ». Si toutefois de grands défis demeurent, cet article reflète une confiance grandissante dans la capacité du gouvernement, des bailleurs de fonds et des partenaires d'exécution à influer sur le taux de prévalence contraceptive moderne, et avec lui, l'avenir de ce pays.

Remerciements : Les auteurs souhaitent remercier Lauren Messina et Kate Schulze, qui ont toutes deux apporté une aide précieuse à l'édition de ce document, ainsi que Awen Southern, qui a traduit cet article de l'anglais au français.

Conflit d'intérêts : Rien à déclarer.

Financement : Les auteurs sont financés par leurs organisations respectives. Cet article n'a pas été commandé à travers des financements d'un seul donateur.

BIBLIOGRAPHIE

1. Profile du pays : République Démocratique du Congo. Site internet de UNdata. http://data.un.org/CountryProfile.aspx?crName=democratic%20republic%20of%20the%20congo. Dernier accès : 18 mai, 2017.
2. Ministère du Plan; Macro International. *Enquête Démographique et de Santé, République Démocratique du Congo 2007.* Calverton, MD: Ministère du Plan and Macro International; 2008. http://dhsprogram.com/pubs/pdf/FR208/FR208.pdf. Dernier accès: 1er février 2018.
3. Ministère du Plan et Suivi de la Mise en óuvre de la Révolution de la Modernité (MPSMRM); Ministère de la Santé Publique (MSP); ICF International. *Enquête Démographique et de Santé en République Démocratique du Congo 2013–2014.* Rockville, MD: MPSMRM, MSP, and ICF International; 2014. http://dhsprogram.com/pubs/pdf/FR300/FR300.pdf. Dernier accès: 1 er février 2018.
4. Barroy H, Andre F, Mayaka S, Samaha H. *Investing in Universal Health Coverage: Opportunities and Challenges for Health Finbancing in the Democratic Republic of Congo.* Washington, DC: World Bank; 2014. https://openknowledge.worldbank.org/handle/10986/23880. Dernier accès: 1er février 2018.
5. Clinton Health Access Initiative (CHAI). Outil de cartographie de suivi de distribution des produits PF en RDC. 21 août 2017.
6. Kayembe P, Babazadeh S, Dikamba N, et al. Family planning supply environment in Kinshasa, DRC: survey findings and their value in advancing family planning programming. *Glob Health Sci Pract.* 2015;3(4):630–645. CrossRef. Medline
7. Mpunga D, Lumbayi JP, Dikamba N, Mwembo A, Mapatano MA, Wembodinga G. Availability and quality of family planning services in the Democratic Republic of Congo: high potential for improvement. *Glob Health Sci Pract.* 2017;5(2):274–285. CrossRef. Medline
8. Bertrand JT, Sullivan TM, Knowles EA, Zeeshan MF, Shelton JD. Contraceptive method skew and shifts in method mix in low- and middle-income countries. *Int Perspect Sex Reprod Health.* 2014;40(3):144–153. CrossRef. Medline
9. Family Planning 2020 (FP2020). FP2020: *Montrer la voie. 2016–2017.* Washington, DC: FP2020; 2017. http://progress.familyplanning2020.org/fr. Dernier accès: 28 février 2018.
10. Ministère de la Santé Publique (MSP). *Planification Familiale: Plan Stratégique National à Vision Multisectorielle (2014–2020).* Kinshasa: MSP; 2014. https://advancefamilyplanning.org/sites/default/files/resources/Final%20Plan%20Strategique%20de%20PF%2020%20Fev%20Ok.pdf. Dernier accès: 1er février 2018.
11. Mukaba T, Binanga A, Fohl S, Bertrand JT. Family planning policy environment in the Democratic Republic of the Congo: levers of positive change and prospects for sustainability. *Glob Health Sci Pract.* 2015;3(2):163–173. CrossRef. Medline
12. Ministère du Plan. *Document de la Stratégie de Croissance et de Réduction de la Pauvreté: Deuxième Génération (2011–2015).* Kinshasa: Ministère du Plan; 2011. https://www.afdb.org/fileadmin/uploads/afdb/Documents/Project-and-Operations/RDC_-_2011-2015_-_Document_de_stratégie_de_réduction_de_la_pauvreté.pdf. 2011. Dernier accès: 23 mars 2017.
13. Advance Family Planning (AFP). La République Démocratique du Congo s'engage pour la planification familiale avec le partenariat mondial PF2020. Baltimore, MD: AFP; 2015. http://advancefamilyplanning.org/sites/default/files/resources/drc_FR.pdf. Dernier accès: 1 er accès 2018.
14. Ministère de la Santé et de l'Hygiène Publique (MSP); Programme National de Santé de l'Adolescent (PNSA). *Plan Stratégique National de la Santé et du Bien-être des Adolescents et des Jeunes 2016–2020.* Kinshasa: MSP et PNSA; 2016.
15. Le Mécanisme de financement mondial a été lancé, avec des milliards de dollars d'ores et déjà mobilisés pour mettre fin à la mortalité maternelle et infantile d'ici 2030 [communiqué de presse]. Banque mondiale; 13 juillet 2015. http://www.banquemondiale.org/fr/news/press-release/2015/07/13/global-financing-facility-launched-with-billions-already-mobilized-to-end-maternal-and-child-mortality-by-2030. Dernier accès: 14 juin 2017.
16. À propos PMA2020. Site internet de Performance Monitoring and Accountability 2020 (PMA2020). http://pma2020.org/about-pma2020. Dernier accès: 24 octobre 2016.
17. Hernandez JH, Akilimali P, Kayembe P, Dikamba N, Bertrand J. The value of spatial analysis for tracking supply for family planning: the case of Kinshasa, DRC. *Health Policy Plann.* 2016;31(8):1058–1068. CrossRef. Medline
18. Performance Monitoring and Accountability 2020 (PMA2020). PMA2013/Kinshasa-R1. Baltimore, MD: PMA2020. https://pma2020.org/sites/default/files/DRC-Kinshasa-R1-EN-FP-Brief-v8-2017-07-13_0.pdf. Dernier accès: 1er février 2018.
19. Performance Monitoring and Accountability 2020 (PMA2020). PMA2020/Kinshasa, DRC: September-November 2017 (Round 6). Baltimore, MD: PMA2020. https://pma2020.org/sites/default/files/PMA2020-Kinshasa-DRC-R6-FP-Brief-En.pdf. Dernier accès: 1er février 2018.
20. Patrick Gold O, Ufuoma John E. Accelerating empowerment for sustainable development: the need for health systems strengthening in Sub-Saharan Africa. *Am J Public Health Res. 2013*;1(7):152–158. CrossRef
21. Tumusiime P, Gonani A, Walker O, Asbu EZ, Awases M, Kariyo PC. Health systems in sub-Saharan Africa: what is their status and role in meeting the health Millennium Development Goals? *Africa Health Monitor.* 2011;(4). http://www.aho.afro.who.int/en/ahm/issue/14/reports/health-systems-sub-saharan-africa-what-their-status-and-role-meeting-health?width=150&height=150. Dernier accès: 1er février 2018.
22. Taux de pénétration de l'internet en 2000-2016. Site internet de l'Union internationale des télécommunications (Genève). Dernier accès: 9 décembre 2017.
23. Democratic Republic of Congo-Telecoms, Mobile and Broadband-Statistics and Analyses. Budde.com website. https://www.budde.com.au/Research/Democratic-Republic-of-Congo-Telecoms-Mobile-and-Broadband-Statistics-and-Analyses. Dernier accès: 19 décembre 2017.
24. Romaniuk A. Persistence of high fertility in tropical Africa: the case of the Democratic Republic of the Congo. *Popul Dev Rev.* 2011;37(1):1–28. CrossRef. Medline
25. Muanda M, Gahungu Ndongo P, Taub LD, Bertrand JT. Barriers to modern contraceptive use in Kinshasa, DRC. *PLoS One.* 2016;11(12):e0167560. CrossRef. Medline
26. Mbadu MM, Ndongo PG, Messina LJ, Bertrand JT. Barriers to modern contraceptive use in rural areas in DRC. *Cult Health Sex.* 2017;19(9):1011–1023. CrossRef. Medline
27. Banque mondiale. DataBank [base de données en ligne]. Washington, DC: Banque mondiale; 2017. https://data.worldbank.org/indicator/NY.GDP.PCAP.CD?locations=CD. Dernier accès: 1er février 2018
28. Programme des Nations Unies pour le développement (PNUD). *Rapport sur le développement humain 2016: Le développement humain pour tous.* New York: PNUD; 2016. http://hdr.undp.org/

sites/default/files/hdr_2016_report_french_web.pdf. Dernier accès: 1er février 2018.

29. Banque mondiale. République Démocratique du Congo- santé, nutrition et population: rapport sur la situation du pays. Washington, DC: Banque mondiale; 2005. http://documents.banquemondiale.org/curated/fr/350801468027254211/Congo-Sante-nutrition-et-population-rapport-analytique-sante-pauvrete. Dernier accès: 31 mai 2017.
30. Population Services International (PSI); FPwatch. DRC 2015 outlet survey findings. FPwatch Research Brief. Washington, DC: PSI; 2016.
31. Organisation mondiale de la santé (OMS). *Everybody's Business: Strengthening Health Systems to Improve Health Outcomes: WHO's Framework for Action*. Genève: OMS; 2007. http://www.who.int/healthsystems/strategy/everybodys_business.pdf?ua=1. Dernier accès: 1er février 2018.
32. Le Partenariat: Qu'est-ce que le Partenariat de Ouagadougou? Site internet de Planification Familiale: Le Partenariat de Ouagadougou. https://partenariatouaga.org/a-propos/le-partenariat/. Dernier accès: 19 décembre 2017.
33. Advance Family Planning (AFP), Institut Bill & Melinda Gates pour la Population et la Santé de la Reproduction, École de Santé Publique Bloomberg, Université Johns Hopkins. *Élaborer une stratégie. AFP SMART: Guide pour l'obtention de Gains Rapides. Établir un consensus, Concentrer les efforts, Réussir le changement.* Baltimore, MD: AFP; 2013. https://advancefamilyplanning.org/sites/default/files/2017-07/AFP%20Portfolio%20Component%202%20FR.pdf. Dernier accès: 19 décembre 2017.
34. Akilimali P, Anglewicz P, Nzuka Engale H, et al.. Differences in family planning outcomes between military and general populations in Kinhsasa, Democratic Republic of Congo: a cross-sectional analysis. 2018. Manuscrit non publié.
35. Binanga A, Bertrand JT. Pilot research as advocacy: the case of Sayana Press in Kinshasa, Democratic Republic of the Congo. *Glob Health Sci Pract.* 2016;4(4):542–551. CrossRef. Medline
36. Bertrand JT, Makani PB, Hernandez J, et al. Acceptability of the community-level provision of Sayana Press by medical and nursing students in Kinshasa, Democratic Republic of the Congo. *Contraception.* 2017;96(3):211–215. CrossRef. Medline
37. Hernandez JH, Mbadu MF, Garcia M, Glover A. The provision of emergency contraception in Kinshasa's private sector pharmacies: experiences of mystery clients. *Contraception.* 2018;97(1):57–61. CrossRef. Medline
38. Hernandez J, Mbadu M, Garcia M. Knowledge, perceptions and attitudes towards emergency contraception among women of reproductive age women in Kinshasa, DRC. *Int Perspect Sex Reprod Health.* À paraître.

Soumis à un comité de lecture

Reçu le : septembre 6, 2017 ; **Accepté le :** janvier 9, 2018

© Kwete et al. Cet article est distribué en accès libre sous les termes de la licence internationale d'attribution de Creative Commons 4.0 (CC BY 4.0), qui permet l'utilisation, la distribution et la reproduction de contenus sans restriction dans tous médias, à condition que l'auteur et la source originale soient dûment cités. Pour lire un exemplaire de la licence, consultez http://creativecommons.org/licenses/by/4.0/. Pour relier cet article, veuillez utiliser le lien permanent suivant: https://doi.org/10.9745/GHSP-D-17-00346-FR
